# *Caenorhabditis elegans* Myotubularin MTM-1 Negatively Regulates the Engulfment of Apoptotic Cells

**DOI:** 10.1371/journal.pgen.1000679

**Published:** 2009-10-09

**Authors:** Wei Zou, Qun Lu, Dongfeng Zhao, Weida Li, James Mapes, Yuting Xie, Xiaochen Wang

**Affiliations:** 1 College of Biological Sciences, China Agricultural University, Beijing, China; 2 National Institute of Biological Sciences, Zhongguancun Life Sciences Park, Beijing, China; 3 Molecular, Cellular, and Developmental Biology, University of Colorado, Boulder, Colorado, United States of America; University of California San Diego, United States of America

## Abstract

During programmed cell death, apoptotic cells are recognized and rapidly engulfed by phagocytes. Although a number of genes have been identified that promote cell corpse engulfment, it is not well understood how phagocytosis of apoptotic cells is negatively regulated. Here we have identified *Caenorhabditis elegans* myotubularin MTM-1 as a negative regulator of cell corpse engulfment. Myotubularins (MTMs) constitute a large, highly conserved family of lipid phosphatases. MTM gene mutations are associated with various human diseases, but the cellular functions of MTM proteins are not clearly defined. We found that inactivation of MTM-1 caused significant reduction in cell corpses in strong loss-of-function mutants of *ced-1*, *ced-6*, *ced-7*, and *ced-2*, but not in animals deficient in the *ced-5*, *ced-12*, or *ced-10* genes. In contrast, overexpression of MTM-1 resulted in accumulation of cell corpses. This effect is dependent on the lipid phosphatase activity of MTM-1. We show that loss of *mtm-1* function accelerates the clearance of cell corpses by promoting their internalization. Importantly, the reduction of cell corpses caused by *mtm-1* RNAi not only requires the activities of CED-5, CED-12, and CED-10, but also needs the functions of the phosphatidylinositol 3-kinases (PI3Ks) VPS-34 and PIKI-1. We found that MTM-1 localizes to the plasma membrane in several known engulfing cell types and may modulate the level of phosphatidylinositol 3-phosphate (PtdIns(3)P) in vivo. We propose that MTM-1 negatively regulates cell corpse engulfment through the CED-5/CED-12/CED-10 module by dephosphorylating PtdIns(3)P on the plasma membrane.

## Author Summary

Clearance of dead cells is crucial for normal animal development. Cell corpses are recognized, engulfed, and removed by phagocytic cells. However, the mechanisms that regulate phagocytosis are still not well understood, especially the ways in which the process is inhibited (negatively regulated). We screened the nematode worm *Caenorhabditis elegans* for negative regulators of cell corpse engulfment and identified myotubularin MTM-1. Myotubularins (MTMs) are a family of highly conserved enzymes that remove phosphate groups from membrane lipids. Mutations in human MTM genes are associated with various severe diseases including X-linked myotubular myopathy and Charcot-Marie-Tooth disease, but the normal functions of MTMs are unknown. In this study, we found that MTM-1 inhibits cell corpse engulfment through a series of evolutionarily conserved signaling molecules (the bipartite GEF (CED-5/DOCK180-CED-12/ELMO) and the GTPase CED-10/Rac). The negative regulatory effect of MTM-1 requires both its lipid phosphatase activity and the function of another group of enzymes called PI3-kinases. We propose that MTM-1 acts through Rac GTPase CED-10 by dephosphorylating the lipid PtdIns(3)P on the plasma membrane. We have identified a negative regulatory mechanism of cell corpse engulfment and a previously unknown cellular function of MTM-1, which may provide further insights into the basis of human MTM-related diseases.

## Introduction

Phagocytosis of apoptotic cells is essential for animal development, tissue homeostasis and regulation of immune responses. Defects in this process contribute to the development of various human diseases including persistent inflammatory diseases and autoimmune disorders [1]. In *C. elegans*, phagocytosis of apoptotic cells is controlled by two partially redundant signaling pathways. In one pathway, three genes, *ced-1*, *ced-7* and *ced-6*, are involved in recognizing and transducing the engulfment signal(s), while *dyn-1* acts downstream of them to promote vesicle delivery for cell corpse internalization [2]–[5]. In the other pathway, several evolutionarily conserved intracellular signaling molecules, CED-2/CrkII, CED-5/Dock180 and CED-12/ELMO, act downstream of PSR-1, the *C. elegans* homologue of the human phosphatidylserine receptor. These signaling molecules mediate activation of the small GTPase CED-10/Rac, leading to rearrangement of the actin cytoskeleton which is needed for cell corpse engulfment [6]–[12]. In addition, CED-10/Rac may also function downstream of the CED-1/6/7 pathway to mediate the engulfment of apoptotic cells [13]. CED-2 belongs to the Crk family whose members are widely expressed adaptor proteins that mediate the formation of protein complexes for signal transduction in response to various extracellular stimuli [14]. However, the way in which CED-2 functions to regulate cell corpse engulfment is not well understood. Biochemical studies in mammalian cells indicate that DOCK180/CED-5 and ELMO/CED-12 function as an unconventional bipartite nucleotide exchange factor for Rac/CED-10 activation, which leads to cytoskeleton reorganization during engulfment [15]. Moreover, an UNC-73/TRIO-MIG-2/RhoG signaling module regulates CED-10/Rac activation through its interaction with the armadillo repeat of CED-12/ELMO [16]. However, no defect in cell corpse engulfment was observed in animals which completely lose the activity of either *mig-2* or *unc-73* or both, indicating that more complex regulatory mechanisms are involved in CED-10/Rac activation. In addition, as earlier studies mainly focused only on positive regulation of engulfment, it is less well understood whether any negative regulatory mechanism is involved.

Myotubularin phosphatases belong to the tyrosine/dual-specificity phosphatase super-family (PTP/DSP) whose members have been found in almost all eukaryotes. Mutations in myotubularin genes are associated with several human diseases [17]. For example, mutations in *MTM1*, the founder member of this family, cause X-linked myotubular myopathy (XLMTM), a severe congenital muscular disorder. Mutations in *MTMR2* and *MTMR13* are associated with Charcot-Marie-Tooth disease (CMT4B1) [18]–[20]. The hallmark of the protein tyrosine phosphatase (PTP) super-family is an active site motif (CX_5_R). Intriguingly, nearly half of the known MTM1-related proteins (MTMRs) carry sequence variations in this motif and are predicted to be catalytically inactive [21]. Nevertheless, these inactive MTMRs are also evolutionarily conserved and have essential biological functions. Recently, several active-inactive pairings of myotubularins have been identified which appear to be important for the function of the active myotubularins, indicating that the inactive MTMRs likely serve as regulatory units for the active ones [22]–[26].

Surprisingly, instead of acting as protein tyrosine phosphatases, MTM1 and its related proteins were later found to function primarily as lipid phosphatases with specificity for phosphatidylinositol 3-phosphate (PtdIns(3)P) and its metabolite phosphatidylinositol 3,5-bisphosphate (PtdIns(3,5)P_2_) [27]–[33]. PtdIns(3)P is mainly generated by the Class III PI3-kinase VPS34 in vivo, and can be further modified by the phosphatidylinositol 3-phosphate 5-kinase PIKFYVE to generate PtdIns(3,5)P_2_ [34]. Both PtdIns(3)P and PtdIns(3,5)P_2_ are key regulators of the endocytic pathway [35]. Therefore, the identification of MTM1 and MTMRs as lipid phosphatases that use PtdIns(3)P and PtdIns(3,5)P_2_ as substrates suggests that they may regulate endocytosis and/or other class III PI3-kinase-mediated cellular events. This idea is supported by evidence from a variety of experiments in yeast and *C. elegans*. Overexpression of human MTM1 decreased the level of PtdIns(3)P in *S. pombe* and induced a vacuolar phenotype similar to that of mutants defective in *VPS34* [33]. Ymr1p, the sole myotubularin in yeast, cooperates with the synaptojanin-like PI phosphatase Sjl3p to regulate PtdIns(3)P levels and vesicular transport [27]. Reduction of *C. elegans mtm-6* activity by RNAi rescued the larval lethality of *vps-34(lf)* mutants, while inactivation of *mtm-1* rescued the endocytosis defect in *vps-34(lf)* coelomocytes (the specialized cells that constantly take up fluid and macromolecules from the body cavity), indicating that myotubularins likely modulate VPS-34-mediated cellular processes in worms [36]. In addition, MTM-6, an active MTM, was found to act together with MTM-9, a catalytically inactive MTM, to regulate an ARF-6 and RME-1-mediated endocytic pathway [24]. However, it is not known how *C. elegans* MTM-1 regulates endocytosis or whether it functions in other cellular processes by regulating PtdIns(3)P.

On the other hand, the cellular function of MTM1 in mammals appears to be more obscure. In one study, it was reported that MTM1 translocates to late endosomes after EGF stimulation and negatively regulates EGFR degradation and vesicular formation at the late stage of endosomal trafficking [37]. This process is mediated through interaction between the MTM1 PH-GRAM domain and PtdIns(3,5)P_2_ [37]. In another study, however, MTM1 siRNA caused increased PtdIns(3)P levels and accumulation of EGFR in early but not late endosomes, whereas MTMR2 RNAi resulted in elevated PtdIns(3)P levels and EGFR accumulation in late endosomes [38]. This suggested that MTM1 and MTMR2 function sequentially in the endocytic pathway with MTM1 acting in an early step [38]. Therefore, the cellular functions of MTM1 either in endocytic transport or in other cellular events still remain elusive.

In the present study, we have identified *C. elegans* MTM-1 as a negative regulator of cell corpse engulfment. We found that inactivation of MTM-1 by RNAi promotes cell corpse engulfment, whereas overexpression of MTM-1 results in accumulation of cell corpses in a manner dependent on its lipid phosphatase activity. We show that the reduction of cell corpses caused by *mtm-1* RNAi requires the functions of CED-5, CED-12 and CED-10 and the activities of PI3-kinases VPS-34 and PIKI-1. MTM-1 is widely expressed in many cell types, localizes to the plasma membrane and negatively regulates the vesicular accumulation of PtdIns(3)P in *C. elegans*. Our data suggest that MTM-1 acts as a lipid phosphatase to negatively regulate cell corpse engulfment through the CED-5/CED-12/CED-10 module, which is likely achieved by dephosphorylating PtdIns(3)P on the plasma membrane.

## Results

### Isolation of *mtm-1* as a negative regulator of cell corpse engulfment

**Figure 1:**
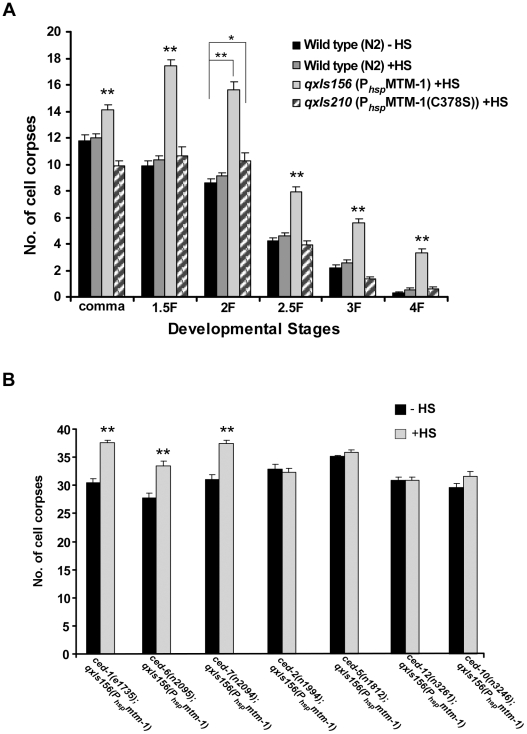
Overexpression of MTM-1 results in accumulation of cell corpses. (A) Time-course analysis was performed in wild type (no heat-shock treatment: black; after heat-shock treatment: gray), *qxIs156* (P*_hsp_*MTM-1; light gray) and *qxIs210* (P*_hsp_*MTM-1(C378S); hatching). Cell corpses were scored at the following embryonic stages: bean/comma (comma), 1.5-fold (1.5F), 2-fold (2F), 2.5-fold (2.5F), 3-fold (3F) and 4-fold (4F). At least 15 embryos were scored at every stage; error bars indicate s.e.m. Data derived from wild type with and without heat-shock treatment, wild type and *qxIs156* or wild type and *qxIs210* at multiple developmental stages were compared by two-way analysis of variance. Post-hoc comparisons were done by Fisher's PLSD (protected least squares difference). *P<0.05, **P<0.0001. All other points had *P* values>0.05. (B) Cell corpses in the indicated strains were scored at the 4-fold embryonic stage. At least 15 embryos were scored; error bars indicate s.e.m. Data were compared by unpaired *t* tests. **P<0.0001; other points had *P* value>0.05. In (A) and (B), heat-shock experiments were performed as described in Materials and Methods.

To identify negative regulators of cell corpse engulfment, we performed an RNAi screen to search for genes which when inactivated cause a reduction in the number of cell corpses in *ced-1(e1735)* mutants (Materials and Methods). *mtm-1* was one of the candidate genes identified from this screen because the accumulation of cell corpses in *ced-1(e1735);rrf-3(pk1426)* double mutants was significantly reduced when treated with *mtm-1* RNAi (Table 1; Materials and Methods). Interestingly, a similar reduction in cell corpses was also observed in strong loss-of-function mutants of *ced-6*, *ced-7*, *ced-2* after *mtm-1* RNAi treatment, but not in animals with mutations in the *ced-5*, *ced-12* or *ced-10* genes (Table 1). The RNAi treatment likely caused a specific inactivation of the *mtm-1* gene as injection of an in vitro-synthesized dsRNA of *mtm-1* resulted in a similar reduction of cell corpses in *ced-1(e1735)* mutants (Figure S1A; Materials and Methods). Moreover, MTM-1::GFP expression was greatly reduced after *mtm-1* RNAi treatment, either by injecting in vitro-synthesized *mtm-1* dsRNA or by feeding with bacteria expressing *mtm-1* dsRNA (Figure S1A). To further confirm the RNAi results, we also analyzed an *mtm-1* deletion mutant, *ok742*, which contains a 1314 bp deletion that removes the region from intron 4 to exon 7 of the *mtm-1* gene (Figure S1B). *ok742* causes embryonic lethality and early larval arrest, indicating that *mtm-1* is essential for embryonic and larval development in *C. elegans*. We examined the appearance of cell corpses in *ok742* homozygous embryos derived from *ok742/+* mothers (*ht2/mtm-1(ok742)*) and found that it was indistinguishable from that in wild-type embryos (Table 1). However, consistent with the RNAi results, *mtm-1(ok742)* deletion mutants caused significant reduction in cell corpses in *ced-2(n1994)* mutants, but not in *ced-5(n1812)* or *ced-10(n3246)* mutants (Table 1). Moreover, similar reduction of cell corpses by *mtm-1* RNAi was also observed in *ced-1(e1735);ced-2(n1994)* double mutants, but not in *ced-1(e1735);ced-5(n1812)*, *ced-1(e1735)ced-12(n3261)* or *ced-1(e1735);ced-10(n3246)* double mutants (Table 1). These data suggest that *mtm-1* likely antagonizes cell corpse engulfment in a way that requires the activity of *ced-5*, *ced-12* and *ced-10*. In agreement with this notion, overexpression of MTM-1 driven by *C. elegans* heat-shock promoters (*qxIs156*: P*_hsp_mtm-1*) led to accumulation of cell corpses at every embryonic stage (Figure 1A). The engulfment defect caused by mutations in the *ced-2*, *ced-5*, *ced-12* or *ced-10* genes was not further enhanced by overexpression of MTM-1, whereas significantly more cell corpses were observed in loss-of-function mutants of *ced-1*, *ced-6* and *ced-7* which overexpressed MTM-1, indicating that MTM-1 negatively regulates cell corpse engulfment and likely acts through the CED-5/CED-12/CED-10 module (Figure 1B). Since *mig-2* modulates the CED-10/Rac pathway through CED-12/CED-5 GEF and seems to act in parallel to *ced-2* [16], we tested whether loss of *mtm-1* function could reduce cell corpses when *mig-2* function was lost. We found that *mtm-1* RNAi resulted in reduced numbers of cell corpses in both *ced-1(e1735);mig-2(mu28)* and *ced-2(n1994);mig-2(mu28)* double mutants, suggesting that an additional input of the CED-10/Rac pathway in parallel to *mig-2* and *ced-2* may exist and *mtm-1* probably functions downstream of them to inhibit the CED-5/CED-12/CED-10 module (Table 1). Consistent with this idea, we observed a significant reduction in cell corpses by *mtm-1* RNAi in a weak loss-of-function mutant of *ced-10*, *ced-10(n1993)*, in which *ced-10* activity is only partially blocked (Table 1).

**Table 1:** *mtm-1* negatively regulates cell corpse engulfment through *ced-5/12/10*.

Genotype	No. of cell corpses^5^	Changes	*p*- value^6^
Wild type	0.3±0.1		
*mtm-1(ok742)* 1	0.4±0.1	No	0.7
*rrf-3(pk1426);control RNAi*	0.6±0.1		
*rrf-3(pk1426);mtm-1 RNAi*	0.5±0.1	No	0.6
*ced-1(e1735);control RNAi* 2	31.8±0.8		
*ced-1(e1735);mtm-1 RNAi* 2	17.1±0.8	Reduced	<0.0001
*ced-6(n2095);control RNAi* 2	31.2±0.7		
*ced-6(n2095);mtm-1 RNAi* 2	19.2±1.2	Reduced	<0.0001
*ced-6(qx17);control RNAi*	13.7±0.6		
*ced-6(qx17); mtm-1 RNAi*	5.7±0.6	Reduced	<0.0001
*ced-7(n2094);control RNAi* 2	29.9±0.8		
*ced-7(n2094);mtm-1 RNAi* 2	23.8±0.8	Reduced	<0.0001
*ced-2(n1994);control RNAi* 2	29.0±0.8		
*ced-2(n1994);mtm-1RNAi* 2	15.8±0.8	Reduced	<0.0001
*ced-2(n1994)* 3	29.3±0.6		
*mtm-1(ok742);ced-2(n1994)* 1 ^,^ 3	17.3±0.7	Reduced	<0.0001
*ced-5(n1812);control RNAi* 2	33.7±0.7		
*ced-5(n1812);mtm-1 RNAi* 2	33.4±1.2	No	0.8
*ced-5(n1812)* 3	32.3±0.6		
*mtm-1(ok742); ced-5(n1812)* 1 ^,^ 3	32.6±0.7	No	0.8
*ced-12(n3261);control RNAi* 2	29.9±0.6		
*ced-12(n3261);mtm-1 RNAi* 2	30.2±0.7	No	0.8
*ced-10(n3246);control RNAi*	29.4±0.5		
*ced-10(n3246);mtm-1 RNAi*	28.2±0.8	No	0.8
*ced-10(n1993);control RNAi*	16.6±0.6		
*ced-10(n1993);mtm-1 RNAi*	11.7±0.9	Reduced	<0.0001
*ced-10(n3246)* 3	28.4±0.5		
*mtm-1(ok742);ced-10(n3264)* 1 ^,^ 3	28.2±0.5	No	0.2
*ced-1(e1735);ced-2(n1994);control RNAi*	44.6±0.9		
*ced-1(e1735);ced-2(n1994);mtm-1 RNAi*	34.1±1.2	Reduced	<0.0001
*ced-1(e1735);ced-5(n1812);control RNAi*	42.7±0.9		
*ced-1(e1735);ced-5(n1812);mtm-1 RNAi*	42.9±0.8	No	0.8
*ced-1(e1735) ced-12(n3261);control RNAi* 4	42.7±1.2		
*ced-1(e1735) ced-12(n3261);mtm-1 RNAi* 4	43.4±1.1	No	0.6
*ced-1(e1735);ced-10(n3246);control RNAi*	45.1±0.8		
*ced-1(e1735);ced-10(n3246);mtm-1 RNAi*	43.7±0.6	No	0.2
*ced-1(e1735);mig-2(mu28);control RNAi*	37.7±0.4		
*ced-1(e1735);mig-2(mu28);mtm-1 RNAi*	31.4±0.6	Reduced	<0.0001
*ced-2(n1994);mig-2(mu28);control RNAi*	39.6±0.7		
*ced-2(n1994);mig-2(mu28);mtm-1 RNAi*	30.2±0.6	Reduced	<0.0001

RNAi experiments were performed as described in Materials and Methods.

1 *mtm-1(ok742)* is balanced by hT2 and non-green progeny were scored as *mtm-1(ok742)* homozygotes.

2 Strains also carry *rrf-3(pk1426)*.

3 Strains were kept on NGM plates seeded with OP50.

4 Strains also carry *unc-101(m1)*.

5 Cell corpses were scored in the head region of 4-fold stage embryos and are shown as mean±s.e.m. At least 15 embryos were scored for each strain.

6 Unpaired *t* tests were performed to compare the average number of cell corpses in *mtm-1* RNAi-treated animals with that in control animals, or to compare the number of cell corpses in double mutants with that in the respective single mutants.

### Loss of *mtm-1* function promotes the internalization of cell corpses

**Figure 2:**
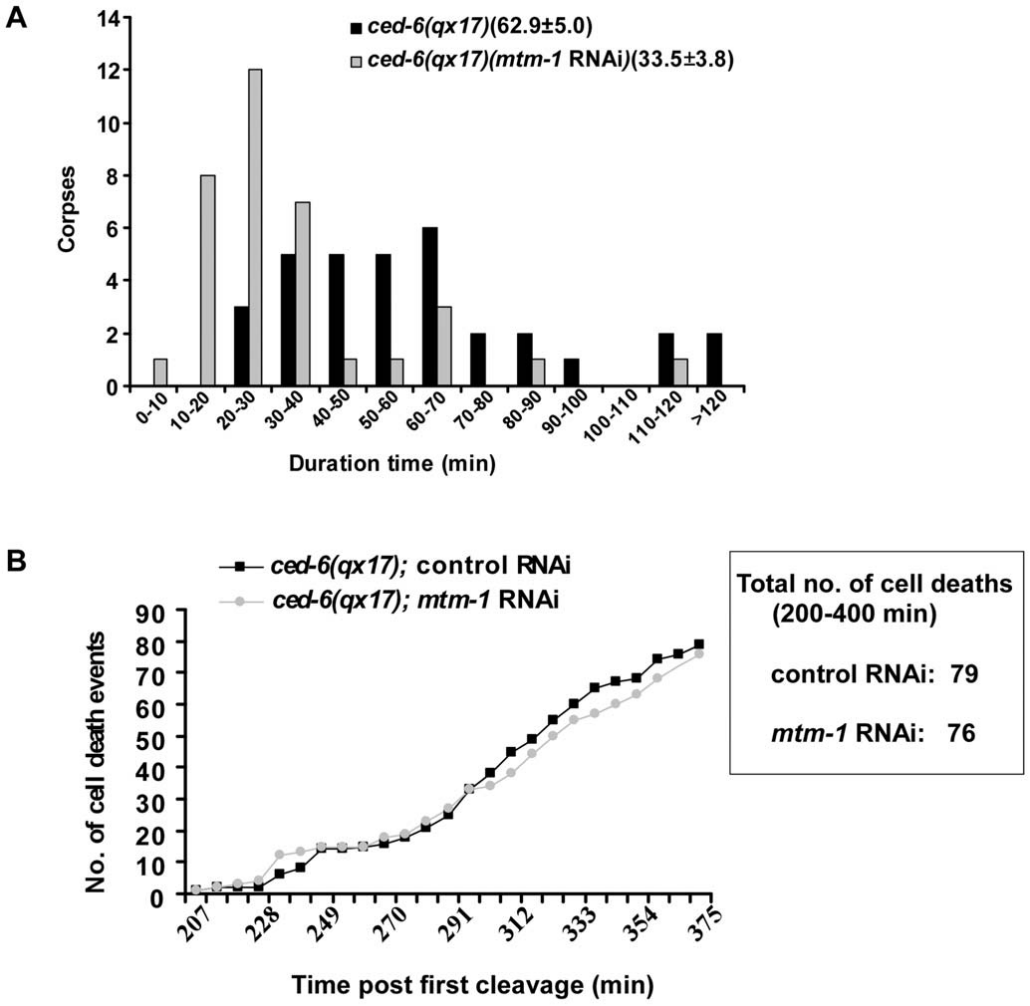
Inactivation of MTM-1 accelerates cell corpse clearance. (A) Four-dimensional microscopy analysis of cell corpse duration was performed in *ced-6(qx17)* mutants (black) or *ced-6(qx17);mtm-1*(*RNAi*) worms (gray). The duration of 33 cell corpses in *ced-6(qx17)* animals (n = 4) and 35 cell corpses in *ced-6(qx17);mtm-1*(*RNAi*) animals (n = 3) was monitored. The numbers in parenthesis indicate the average duration of cell corpses (±s.e.m). The *y*-axis represents the number of cell corpses within a specific duration range as shown on the *x*-axis. (B) *mtm-1* RNAi does not affect cell death occurrence. Embryonic cell deaths occurring between 200 and 400 min post first cleavage were followed in *ced-6(qx17)* mutants treated with either control RNAi (black) or *mtm-1* RNAi (gray). The *y*-axis indicates the total number of cell death events observed at different time points as shown on the *x*-axis.

To further investigate whether the reduction in cell corpses caused by inactivation of *mtm-1* is due to an accelerated clearance of cell corpses, we performed a time-lapse analysis to follow cell corpse duration in *qx17*, a weak loss-of-function allele of the *ced-6* gene which showed a mild engulfment defect on its own and a reduced number of cell corpses after *mtm-1* RNAi treatment (Table 1; Materials and Methods). In *ced-6(qx17)* animals treated with control RNAi, most cell corpses persisted from 30 to 70 min, with an average duration of 63 min (Figure 2A). In contrast, in *ced-6(qx17)* embryos treated with *mtm-1* RNAi, most cell corpses persisted from 20 to 40 min, with an average duration of 34 min, which is 46% shorter than in control animals (Figure 2A). This suggests that *mtm-1* RNAi may promote cell corpse clearance. We next examined the embryonic cell deaths that occurred during a period of 200–400 min past the first cleavage. We observed similar numbers of cell death events in *qx17* animals treated with either control or *mtm-1* RNAi, indicating that *mtm-1* RNAi does not obviously affect the occurrence of cell death (Figure 2B). Consistent with this, no missing or extra cells were observed in the anterior pharynx of *ced-6(qx17)* animals treated with either control or *mtm-1* RNAi (data not shown). Therefore, inactivation of *mtm-1* accelerates the clearance of cell corpses.

**Figure 3:**
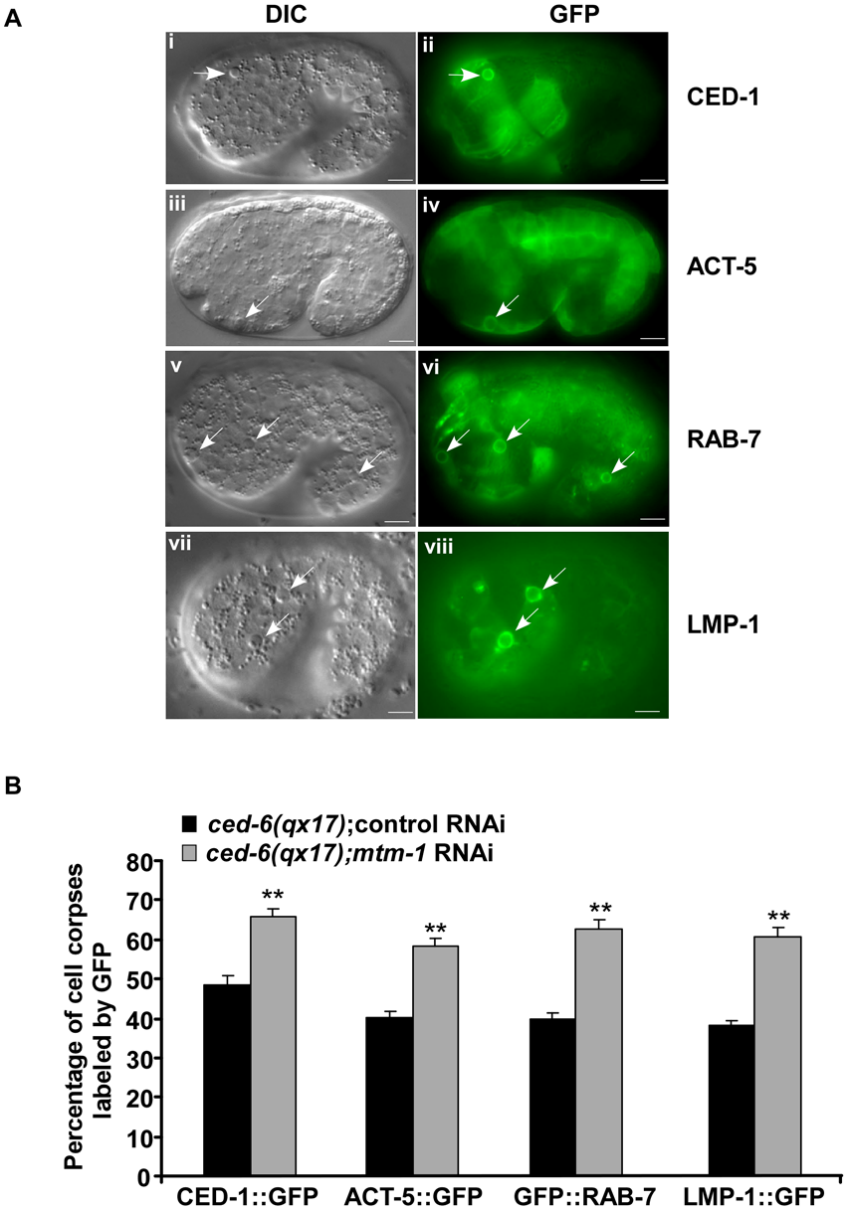
Loss of *mtm-1* function promotes the internalization of cell corpses. (A) DIC and fluorescence images of wild-type embryos expressing CED-1::GFP (i, ii), ACT-5::GFP (iii, iv), GFP::RAB-7 (v, vi) or LMP-1::GFP (vii, viii) are shown. Cell corpses surrounded by GFP are indicated by arrows. Bars, 5 µm. (B) The percentage of cell corpses encircled by CED-1::GFP, ACT-5::GFP, GFP::RAB-7 or LMP-1::GFP was quantified in *ced-6(qx17)* embryos treated with either control (black bar) or *mtm-1* RNAi (gray bar). At least 17 1.5-fold stage embryos were scored; error bars indicate s.e.m. Unpaired *t* tests were performed to compare the data. **P<0.0001.

**Figure 4:**
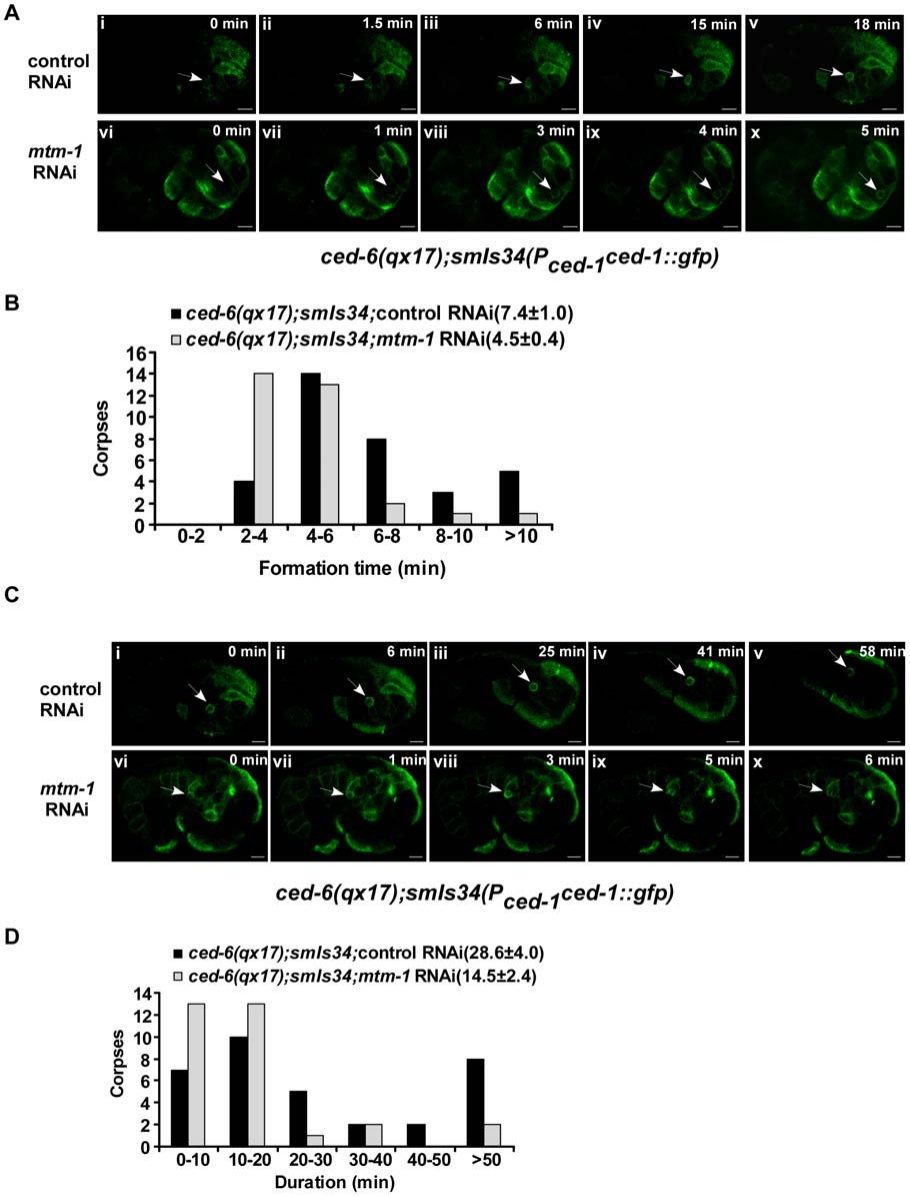
*mtm-1* RNAi accelerates the engulfment of cell corpses. (A) The formation of CED-1::GFP rings around cell corpses was followed and confocal time-lapse images of *ced-6(qx17);smIs34(*P*_ced-1_ced-1::gfp*) embryos treated with either control (i–v) or *mtm-1* RNAi (vi–x) are shown. The time point immediately prior to the appearance of trace amounts of CED-1::GFP adjacent to each cell corpse (arrowed) was set as 0 min. Bars: 5 µm. (B) Quantification of data shown in (A). 34 and 31 cell corpses were followed in control and *mtm-1* RNAi-treated embryos, respectively. Numbers in parenthesis indicate the average formation time of the CED-1::GFP ring (±s.e.m). (C) The duration of CED-1::GFP around each cell corpse was followed and confocal time-lapse images of *ced-6(qx17);smIs34*(P*_ced-1_ced-1::gfp*) embryos treated with either control (i–v) or *mtm-1* RNAi (vi–x) are shown. The first time point (0 min) was set when a full CED-1::GFP ring (arrowed) was just seen. Bars: 5 µm. (D) Quantification of data shown in (C). 34 and 31 cell corpses were quantified in control and *mtm-1* RNAi-treated embryos, respectively. Numbers in parenthesis indicate the average duration time of the CED-1::GFP ring (±s.e.m).

*ced-1* encodes a transmembrane phagocytic receptor that acts specifically in engulfing cells to mediate the recognition and internalization of cell corpses [2]. In wild-type animals, CED-1::GFP was detected along the surface of apoptotic cells, which are distinguishable using Nomarski optics [2],[5] (Figure 3A; Figure S2A). Moreover, we found that clustering of CED-1::GFP around cell corpses completely overlapped with a secreted Annexin V::mRFP fusion protein expressed under the control of heat-shock promoters (P*_hsp_annexin v::mrfp*) (Figure S2A). Annexin V specifically labels apoptotic cells by binding to phosphatidylserine (PS), an “eat me” signal which appears only on the surface of dying cells [39]–[41]. In fact, over 96% of cell corpses were found to be labeled by the secreted Annexin V::mRFP in both wild type and *ced-6(qx17)* mutants (at least 15 embryos were scored in each strain). We therefore used CED-1::GFP as a marker to examine whether the acceleration of cell corpse clearance caused by *mtm-1* RNAi is achieved by facilitating the internalization of apoptotic cells. We found that more cell corpses were surrounded by CED-1::GFP in *ced-6(qx17)* animals treated with *mtm-1* RNAi, suggesting that more apoptotic cells might be internalized (Figure 3B). A similar increase in CED-1::GFP clustering by *mtm-1* RNAi was also observed in the strong loss-of-function mutants of *ced-6* and *ced-2*, but not *ced-5* (Figure S3A). Since CED-1 is a phagocytic receptor that localizes to extending pseudopods and only transiently associates with nascent phagosomes after engulfment [2],[5], we further examined the internalization of cell corpses by monitoring both the formation and duration of the CED-1::GFP ring around cell corpses. We found that in *ced-6(qx17)* animals treated with *mtm-1* RNAi, the CED-1::GFP ring formed rapidly around dying cells, in an average of 4.5 min (Figure 4A and 4B; Video S1). In control RNAi-treated *ced-6(qx17)* embryos, however, many cell corpses were not fully surrounded by CED-1::GFP even after 10 min and the average formation time of a full CED-1::GFP ring was 7.4 min, which is 64% longer than that in *mtm-1* RNAi-treated embryos (Figure 4A and 4B; Video S2). Moreover, we observed that CED-1::GFP associated with extending pseudopods or nascent phagosomes for an average duration of 29 min in control animals, compared to an average of just 15 min in *ced-6(qx17);mtm-1(RNAi)* embryos (Figure 4C and 4D; Video S3, Video S4). To exclude the possibility that the observed effect in cell corpse engulfment by *mtm-1* RNAi is caused by the individual variability of different apoptotic cells or different developmental locations, we also monitored the clearance of a specific apoptotic cell C3, which undergoes apoptosis at a mid-embryonic stage and is engulfed by a ventral hypodermal cell [5],[42] (Figure S2A). We found that both formation and duration of the CED-1::GFP ring around C3 were significantly shortened after *mtm-1* RNAi treatment, indicating that the internalization of C3 was accelerated similar to other apoptotic cells (Figure S2B, S2C). Collectively, these data suggest that inactivation of MTM-1 promotes cell corpse internalization in *ced-6(qx17)* mutants.

To further prove that *mtm-1* RNAi promotes the engulfment of cell corpses, we examined the internalization of apoptotic cells by using a GFP fusion of a cytosolic actin isoform, ACT-5, which clusters around cell corpses during early stages of engulfment and disappears after they are fully engulfed [13]. We found that more cell corpses were labeled by ACT-5::GFP in *ced-6(qx17)* embryos treated with *mtm-1* RNAi than in control animals (Figure 3). A similar increase in the labeling of cell corpses was also observed in *ced-6(qx17);mtm-1(RNAi)* embryos when two different phagosomal markers, GFP::RAB-7 and LMP-1::GFP, were monitored (Figure 3). RAB-7 associates with phagosomal membranes and controls late steps of phagosome maturation, while LMP-1, a lysosome-associated membrane protein, is recruited to phagosomes during late stages of phagosome maturation [43]–[46]. Therefore, our data suggest that more cell corpses are internalized and enclosed in phagosomes when *mtm-1* function is inhibited. As expected, the clustering of both GFP::RAB-7 and LMP-1::GFP around cell corpses was significantly enhanced in *ced-1(e1735)*, *ced-6(n2095)* and *ced-2(n1994)* mutants but not *ced-5(n1812)* mutants after *mtm-1* RNAi treatment (Figure S3B, S3C). This supports the hypothesis that *mtm-1* negatively regulates cell corpse engulfment through *ced-5*/*12/10*.

### MTM-1 does not play a similar role in the migration of distal tip cells

Mutations in *ced-2*, *ced-5*, *ced-12* and *ced-10*, but not *ced-1*, *ced-6* or *ced-7*, affect the migration of the two distal tip cells (DTCs), which are located at the tips of the two gonad arms and guide the formation of gonads during larval development [8],[10],[11],[47],[48]. In *ced-2*, *ced-5*, *ced-12* and *ced-10* mutants, the DTCs often make extra turns, which causes abnormally shaped gonads [8],[10],[11]. Since *mtm-1* negatively regulates cell corpse engulfment through the CED-5/CED-12/CED-10 complex and both MTM-1 and CED-10 are highly expressed in DTCs ([49] and see below), we tested whether *mtm-1* could also modify the DTC migration defect in *ced-2*, *ced-5*, *ced-12* or *ced-10* mutants. We found that *mtm-1* RNAi did not obviously affect the DTC migration defect in strong loss-of-function mutants of *ced-2*, *mig-2*, *ced-5*, *ced-12* or *ced-10*, nor did it significantly suppress or enhance the abnormal DTC migration phenotype in *ced-2;mig-2* or *ced-1;ced-2* double mutants (Table S1). However, we observed that *mtm-1* RNAi resulted in a weak DTC migration defect in both wild type and *rrf-3(pk1426)* mutants (which are hypersensitive to RNAi treatment). Moreover, inactivation of *mtm-1* by RNAi significantly enhanced the defect of DTC migration in *ced-10(n1993)* weak loss-of-function mutants (Table S1). This suggests that *mtm-1* may play a positive role in the migration of distal tip cells and may act in the same genetic pathway as *ced-10*.

### Other MTMs do not play redundant roles with MTM-1 in cell corpse engulfment

MTM-1 belongs to the myotubularin family, which constitutes a large group within the tyrosine/dual-specificity phosphatase (PTP/DSP) super-family and which are evolutionarily conserved in yeast, worms and humans [17] (Figure S4). To examine whether the function of MTM-1 in cell corpse engulfment is conserved, we overexpressed human MTM1 under the control of the *C. elegans* heat-shock promoters (P*_hsp_*hMTM1) and found that it efficiently rescued the reduced cell corpse phenotype in *mtm-1(ok742);ced-2(n1994)* mutants (Table S2). This suggests that human MTM1 can substitute for the function of worm MTM-1 in regulating cell corpse engulfment.

In *C. elegans*, 5 MTMs have been identified based on sequence homology [36]. Like mammalian MTMs, they may have non-redundant functions. To determine whether other *C. elegans* MTMs are also involved in cell corpse clearance, we analyzed the cell corpse phenotype of *mtm(lf)* in the background of *ced-1(e1735)*, which has a cell corpse phenotype that can be attenuated by *mtm-1* RNAi. We found that the persistent cell corpse phenotype of *ced-1(e1735)* mutants was not affected when *mtm-9*, *mtm-6*, or *mtm-5* was inactivated either individually or in combination (Table S3). Interestingly, when *mtm-3* was inactivated by RNAi in *ced-1(e1735)* mutants, we observed a slight enhancement of cell corpse numbers which was further enhanced by *mtm-6(ok330)* but not *mtm-5(ok469)* mutants (Table S3). In wild-type animals, *mtm-3* RNAi also caused increased cell corpse numbers, which were significantly enhanced by *mtm-6(ok330)* but not *mtm-5(ok469)* mutants (Table S3). This suggests that *mtm-3* may play a redundant role with *mtm-6* to promote cell corpse clearance or to affect cell death activation. However, since *mtm-1* RNAi treatment resulted in an opposite phenotype in *ced-1(e1735)* mutants, these MTMs appear not to act redundantly with MTM-1 in cell corpse engulfment.

### MTM-1 acts as a lipid phosphatase to regulate cell corpse engulfment

Although myotubularins contain CX_5_R active site motifs characteristic of the protein tyrosine phosphatase super-family, they primarily function as lipid phosphatases to dephosphorylate phosphatidylinositol 3-phosphate (PtdIns(3)P) or phosphatidylinositiol 3, 5-bisphosphate (PtdIns(3,5)P_2_) [28],[29],[32]. To determine whether MTM-1 acts as a lipid phosphatase to regulate cell corpse engulfment, we generated a catalytically inactive mutant of MTM-1, MTM-1(C378S) [50]. In contrast to overexpression of MTM-1, overexpression of MTM-1(C378S) driven by the *C. elegans* heat-shock promoters (P*_hsp_*MTM-1(C378S)) failed to cause accumulation of cell corpses and was unable to rescue the reduced cell corpse phenotype in *mtm-1(ok742);ced-2(n1994)* double mutants (Figure 1A; Table S2). This indicates that the lipid phosphatase activity of MTM-1 is required for its function in cell corpse engulfment.

**Figure 5:**
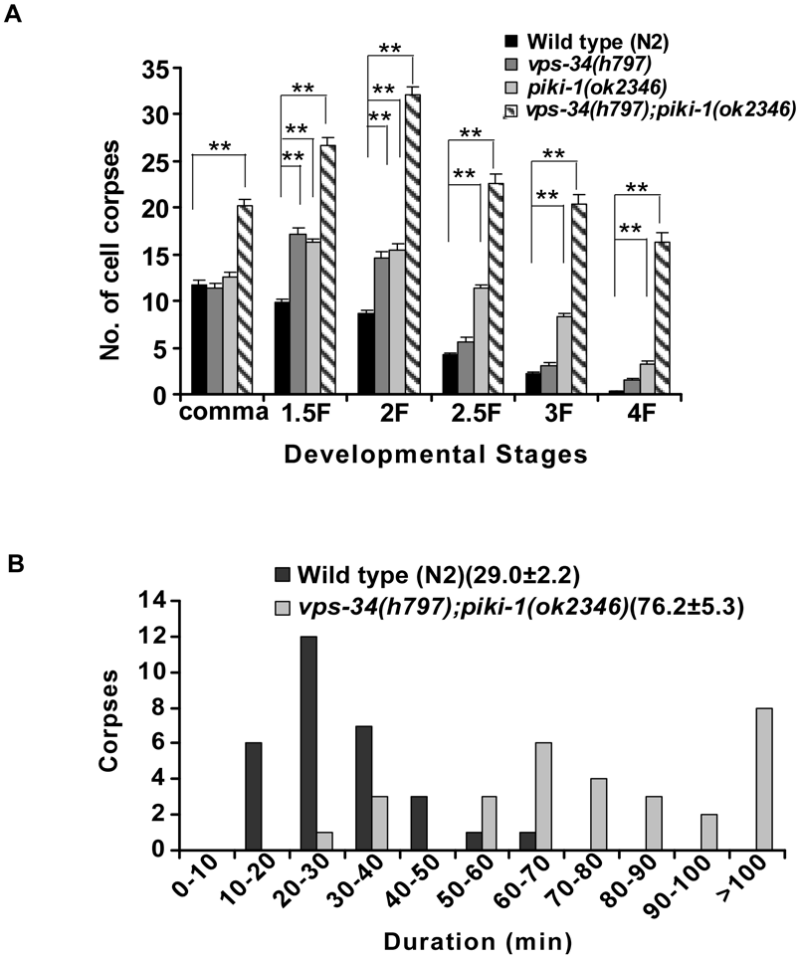
PI3Ks VPS-34 and PIKI-1 are important for cell corpse clearance. (A) PI3Ks VPS-34 and PIKI-1 act redundantly to remove apoptotic cells. Time-course analysis was performed in wild type (black), *vps-34 (h797)* (gray), *piki-1(ok2346)* (light gray), and *vps-34(h797);piki-1(ok2346)* double mutants (hatching). Cell corpses were scored and analyzed as described in Figure 1. Data from wild type and mutant animals were compared as described in Figure 1. **P<0.0001; all other points had *P* value>0.05. (B) Four-dimensional microscopy analysis of cell corpse duration in *vps-34(h797);piki-1(ok2346)* mutants. The durations of 30 cell corpses from wild type (n = 3; black bars) and 34 cell corpses from *vps-34(h797);piki-1(ok2346)* double mutants (n = 3; gray bars) were monitored as described in Figure 2 and Materials and Methods. The numbers in parenthesis indicate the average duration of cell corpses (±s.e.m).

Since human MTM1 mainly uses PtdIns(3)P as a substrate both in vitro and in vivo [28],[29],[33], we next examined whether phosphatidylinositol 3-kinase (PI3K) activity is required for the reduction of cell corpses caused by *mtm-1* RNAi. Three classes of phosphatidylinositol 3-kinases (PI3K) are responsible for generating 3-phosphoinositides, among which class I PI3Ks are mainly responsible for generating PtdIns(3,4,5)P_3_, while the synthesis of PtdIns(3)P in vivo is mostly carried out by class III PI3Ks [34]. In addition, class II PI3Ks produce PtdIns(3)P both in vitro and in vivo [51],[52]. *C. elegans* contains a single homolog of each class: AGE-1, a class I PI3K essential for the *C. elegans* insulin-like signaling pathway [53]; VPS-34, a class III PI3K that regulates larval development, endocytosis and cell corpse degradation by generating PtdIns(3)P [36],[44],[54]; and a class II PI3-kinase encoded by the open reading frame F39B1.1 which can compensate for the loss of *vps-34* function when *mtm-6* is inactivated by RNAi [36]. We found that the deletion mutant *ok2346* of the class II PI3-kinase F39B1.1, which we named *piki-1* (phosphatidylinositol 3 -kinase), resulted in accumulation of cell corpses at several embryonic stages similar to *vps-34(h797)*, a strong loss-of-function mutant of *vps-34* (Figure 5A; Figure S1C). Interestingly, significantly more cell corpses were observed in *vps-34(h797);piki-1(ok2346)* double mutants than in either single mutant alone (Figure 5A). The increase in cell corpses observed in *vps-34(h797);piki-1(ok2346)* double mutants is likely due to a defect in apoptotic cell clearance because cell corpses persist on average 1.6 times longer in embryos lacking both *vps-34* and *piki-1* activities than in wild type (Figure 5B). This indicates that *vps-34* and *piki-1* act redundantly to regulate the removal of apoptotic cells. In contrast, neither an obvious cell corpse phenotype nor an enhancement of the cell corpse clearance defect was observed when the class I PI3K AGE-1 was inactivated by RNAi in either wild-type animals or *vps-34;piki-1* double mutants, indicating that the class I PI3K is not involved in this process (data not shown).

We next examined whether *mtm-1* RNAi is able to reduce cell corpse numbers in *ced-1(e1735)* mutants when PI3K activity is blocked. Although a reduction in cell corpses was still observed in *vps-34(h797)ced-1(e1735)* or *ced-1(e1735);piki-1(ok2346)* double mutants treated with *mtm-1* RNAi, no obvious difference in the number of cell corpses was seen in *vps-34ced-1;piki-1* triple mutants after *mtm-1* RNAi treatment (Table 2). This indicates that the reduction of cell corpses in *ced-1(e1735)* mutants by *mtm-1* RNAi requires the activity of both class III and class II PI3Ks. Similar effects were also observed when *mtm-1* was inactivated in *vps-34;piki-1* double mutants or in *ced-6* or *ced-7* mutants lacking both *vps-34* and *piki-1* functions (Table 2). Therefore, MTM-1 may coordinate with the PI3Ks VPS-34 and PIKI-1 to regulate the level of PtdIns(3)P for cell corpse engulfment.

**Table 2:** *vps-34* and *piki-1* are required for the reduction in cell corpses by *mtm-1* RNAi.

Genotype	No. of cell corpses^1^	Changes	*p*- value^2^
*vps-34(h797);piki-1(ok2346);control RNAi*	16.3±0.9		
*vps-34(h797);piki-1(ok2346);mtm-1 RNAi*	14.7±1.2	No	0.3
*vps-34(h797)ced-1(e1735);control RNAi*	33.6±0.8		
*vps-34(h797)ced-1(e1735);mtm-1 RNAi*	22.6±1.0	Reduced	<0.0001
*ced-1(e1735);piki-1(ok2346);control RNAi*	36.9±0.5		
*ced-1(e1735);piki-1(ok2346);mtm-1 RNAi*	25.9±1.0	Reduced	<0.0001
*vps-34(h797)ced-1(e1735);piki-1(ok2346);control RNAi*	46.7±0.5		
*vps-34(h797)ced-1(e1735);piki-1(ok2346);mtm-1 RNAi*	47.5±0.8	No	0.4
*vps-34(h797);ced-6(n2095);control RNAi*	38.4±0.6		
*vps-34(h797)ced-6(n2095);mtm-1 RNAi*	31.6±0.7	Reduced	<0.0001
*ced-6(n2095);piki-1(ok2346);control RNAi*	37.0±0.7		
*ced-6(n2095);piki-1(ok2346);mtm-1 RNAi*	27.3±0.9	Reduced	<0.0001
*vps-34(h797);ced-6(n2095);piki-1(ok2346);control RNAi*	43.5±0.8		
*vps-34(h797);ced-6(n2095);piki-1(ok2346);mtm-1 RNAi*	44.0±0.5	No	0.6
*vps-34(h797);ced-7(n2094);control RNAi*	32.3±0.5		
*vps-34(h797)ced-7(n2094);mtm-1 RNAi*	23.2±0.8	Reduced	<0.0001
*ced-7(n2094);piki-1(ok2346);control RNAi*	39.1±1.0		
*ced-7(n2094);piki-1(ok2346);mtm-1 RNAi*	32.9±1.6	Reduced	0.004
*vps-34(h797);ced-7(n2094);piki-1(ok2346);control RNAi*	43.5±0.6		
*vps-34(h797);ced-7(n2094);piki-1(ok2346);mtm-1 RNAi*	46.0±0.7	increased	0.009

RNAi experiments were performed as described in Materials and Methods. *vps-34(h797)* mutants were maintained and scored as described in Materials and Methods.

1 Cell corpses were scored in the head region of 4-fold stage embryos as described in Materials and Methods and are shown as mean±s.e.m. At least 15 embryos were scored for each strain.

2 Unpaired *t* tests were performed to compare control animals with *mtm-1* RNAi-treated worms.

### MTM-1 functions in engulfing cells and localizes to the plasma membrane

**Figure 6:**
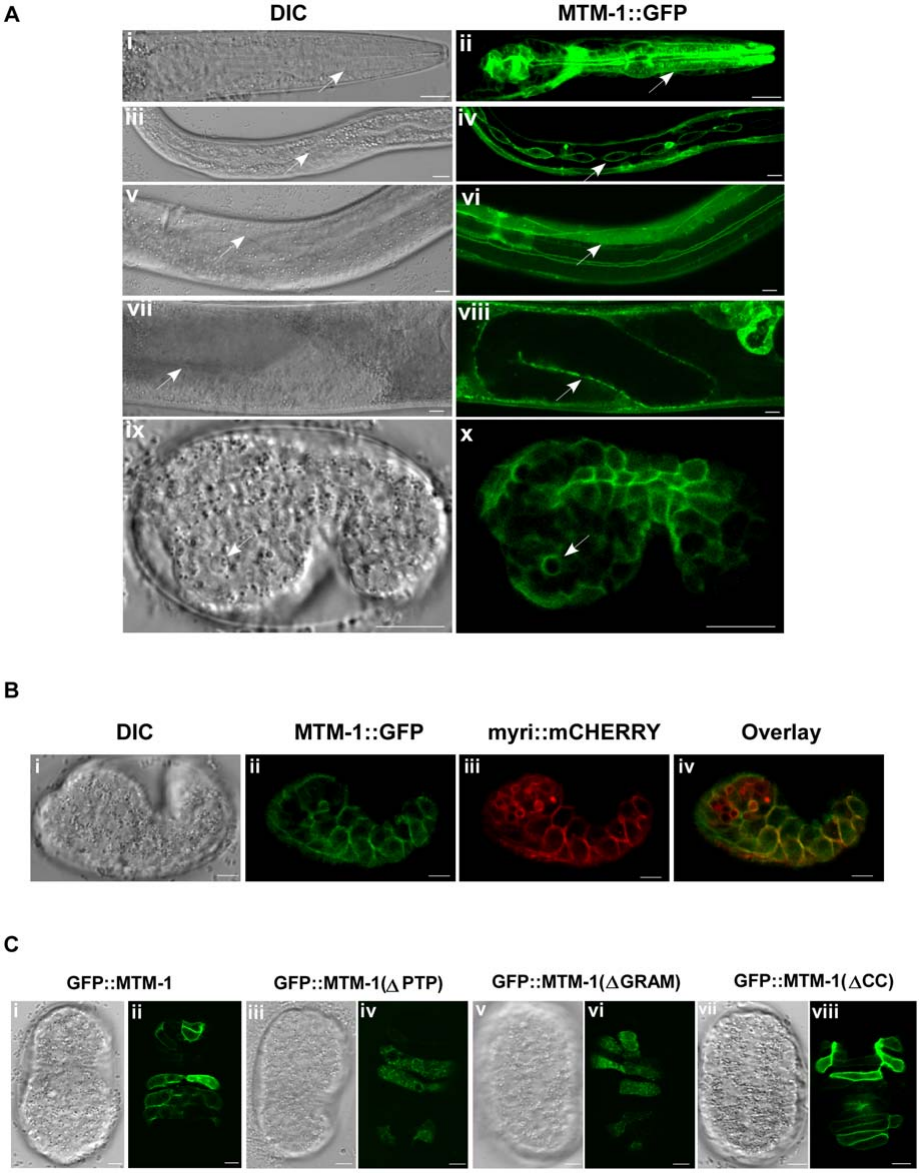
MTM-1 is expressed in engulfing cells and localizes to the plasma membrane. DIC and fluorescent confocal images of wild-type animals carrying an integrated array of P*_mtm-1_*MTM-1::GFP are shown. MTM-1 is expressed in several known engulfing cell types including pharyngeal muscle cells (i, ii), hypodermal cells (iii, iv), body wall muscle cells (v, vi) and sheath cells (vii, viii) as indicated by arrows and is mainly localized to cell membranes. MTM-1::GFP also clusters around apoptotic cells (ix, x). Bars: 10 µm. (B) DIC (i), GFP (ii), mCHERRY (iii) images and the merged image of GFP and mCHERRY (iv) of a wild-type embryo co-expressing MTM-1::GFP driven by *mtm-1* promoter (P*_mtm-1_mtm-1::gfp*) and myri::mCHERRY controlled by heat-shock promoters (P*_hsp_myri::mcherry*) are shown. MTM-1::GFP co-localized with myri::mCHERRY to plasma membranes. Bars: 5 µm. (C) The PH-GRAM and PTP domains of MTM-1 are required for membrane localization of MTM-1. DIC and fluorescent confocal images of full-length (i, ii) and truncated GFP::MTM-1 (iii–viii) controlled by the *ced-1* promoter in wild-type embryos are shown. Membrane localization was clearly seen with full-length MTM-1 (i, ii) and MTM-1(ΔCC) (vii, viii), but not in embryos expressing GFP::MTM-1 lacking either the PTP domain (iii, iv) or the PH-GRAM domain (v, vi). Bars: 5 µm.

To determine the subcellular localization of MTM-1, we generated a MTM-1::GFP fusion driven by the *mtm-1* promoter (P*_mtm-1_mtm-1::gfp*), which fully rescued the cell corpse phenotype of *mtm-1(ok742);ced-2(n1994)* mutants (Table S2). The expression of MTM-1::GFP was seen from embryogenesis throughout larval and adult stages in many known engulfing cell types including hypodermal cells, body wall muscle cells, pharyngeal muscle cells and sheath cells (Figure 6A). MTM-1::GFP was also observed in vulva cells, distal tip cells and coelomocytes, which is consistent with previous findings that *mtm-1* RNAi rescues the coelomocyte uptake defect in *vps-34(lf)* mutants (Figure S5A) [36]. In agreement with this expression pattern, we found that overexpression of MTM-1 controlled by the *ced-1* promoter (P*_ced-1_mtm-1*), which drives gene expression specifically in engulfing cells, fully rescued the reduced cell corpse phenotype in *mtm-1(ok742);ced-2(n1994)* mutants. This rescuing activity was not observed when MTM-1 expression was controlled by the *egl-1* promoter (P*_egl-1_mtm-1*), which drives gene expression specifically in dying cells (Table S2) [2],[55]. This indicates that *mtm-1* needs to function in engulfing cells to regulate cell corpse engulfment.

**Figure 7:**
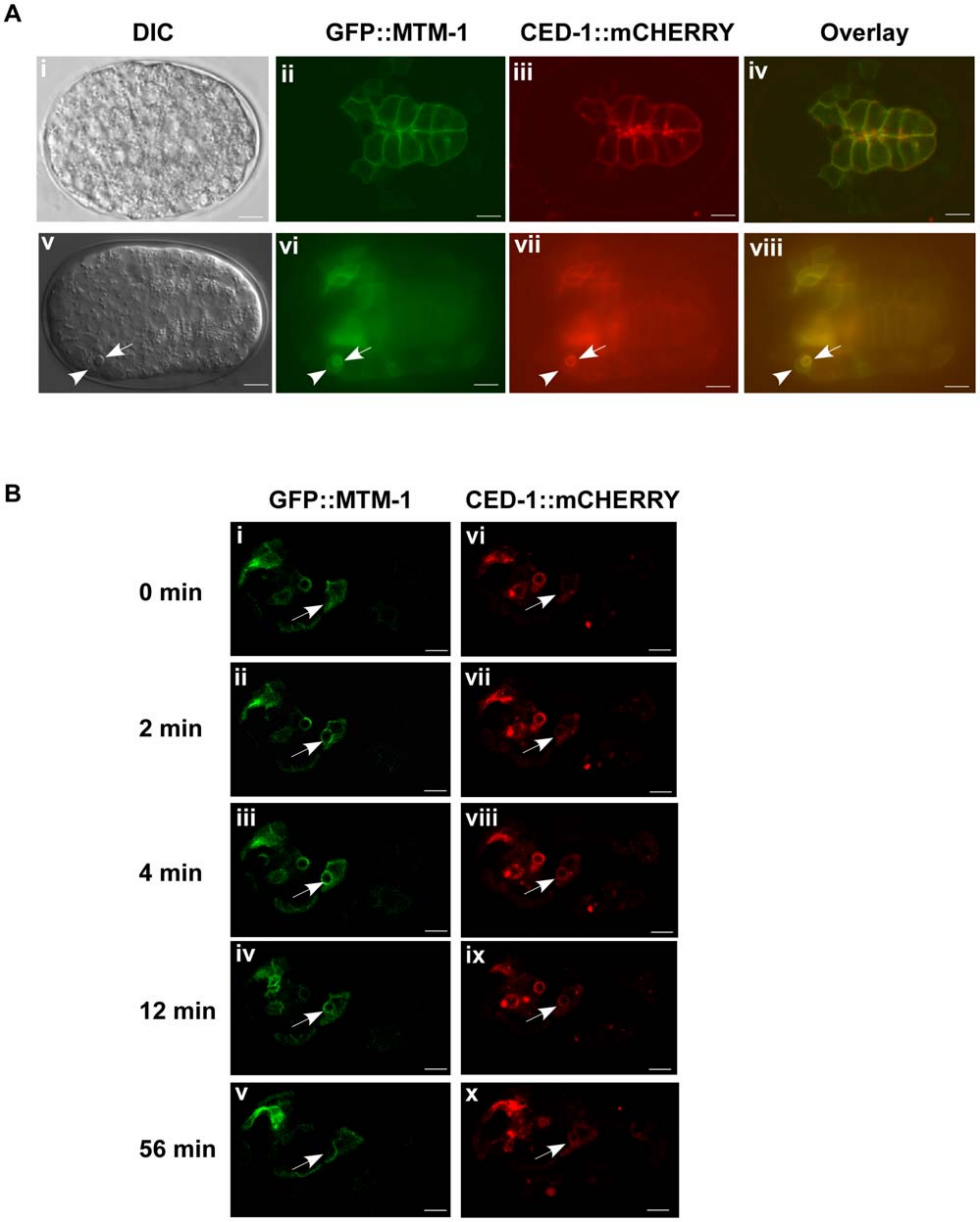
MTM-1 and CED-1 co-localize to apoptotic cells. (A) DIC and fluorescence images of a wild-type embryo expressing both GFP::MTM-1 and CED-1::mCHERRY controlled by the *ced-1* promoter are shown. MTM-1 and CED-1 co-localize to the plasma membrane (i–iv) and are clustered around the same apoptotic cell (arrow) internalized by the neighboring engulfing cell (arrowhead) (v–viii). Bars: 5 µm. (B) MTM-1 transiently associates with the apoptotic cell during engulfment. Confocal time-lapse images of a wild-type embryo co-expressing P*_ced-1_gfp::mtm-1* (i–v) and P*_ced-1_ced-1::mcherry* (vi–x) are shown. The apoptotic cell followed is indicated by the arrow and the time point immediately prior to the appearance of GFP::MTM-1 or CED-1::mCHERRY was set as 0 min. The longer duration of the apoptotic cell is likely caused by overexpression of GFP::MTM-1. Bars: 5 µm.

We found that MTM-1::GFP is mainly localized to the plasma membrane and coincides with myri::mCHERRY, which specifically labels cell membranes (Figure 6; personal communication with Dr. David Sherwood and Dr. Guangshuo Ou; Materials and Methods). GFP::MTM-1 expressed from engulfing cells (P*_ced-1_gfp::mtm-1*) also localizes to plasma membranes and overlaps with CED-1::mCHERRY, a cell surface phagocytic receptor (Figure 7A) [2]. Interestingly, we found that GFP::MTM-1 and CED-1::mCHERRY not only co-localized to the plasma membrane, but clustered around the same apoptotic cell, suggesting that MTM-1 may associate with extending pseudopods or nascent phagosomes at a similar stage to CED-1 (Figure 7A). Indeed, by time-lapse analysis, we observed that GFP::MTM-1 and CED-1::mCHERRY appeared on the surface of dying cells simultaneously during the early stage of engulfment (Figure 7B). However, GFP::MTM-1 disappeared more quickly than CED-1::mCHERRY from the phagosome after internalization, suggesting that MTM-1 only transiently associates with apoptotic cells during engulfment (Figure 7B).

Similar to the mammalian MTM1 protein, *C. elegans* MTM-1 contains several conserved motifs including an N-terminal PH-GRAM domain which may have the capacity to bind phosphoinositides, a central myotubularin-related domain, a protein tyrosine phosphatase domain (PTP) that contains the catalytic activity, and a C-terminal coiled-coil domain (CC) which might be involved in protein-protein interactions [17] (Figure S4). To find out which domain is important for the membrane localization of MTM-1, we generated several truncated GFP::MTM-1 fusions driven by the *ced-1* promoter and examined their localizations. All of the MTM-1 truncations were expressed at the expected size in *C. elegans* (Figure S5B). We found that MTM-1 lacking either the PH-GRAM domain (GFP::MTM-1(ΔGRAM)) or the PTP domain (GFP::MTM-1(ΔPTP)) completely lost its membrane localization and instead displayed a punctate vesicular localization pattern. This indicates that both the PH-GRAM and PTP domains are required for the plasma membrane localization of MTM-1 (Figure 6C). In contrast, GFP::MTM-1(ΔCC), in which the C-terminal coiled-coil motif is deleted, still localized to the plasma membrane (Figure 6C). Moreover, we found that this membrane-localized GFP::MTM-1(ΔCC) was able to rescue the reduced cell corpse phenotype of *mtm-1(ok742);ced-2(n1994)* mutants, suggesting that the coiled-coil motif is dispensable for MTM-1 function in cell corpse engulfment (Table S2). Conversely, neither ΔPH-GRAM nor ΔPTP truncations could rescue the cell corpse phenotype of *mtm-1* deletion mutants (Table S2).

Since the PH-GRAM domain likely mediates the binding of myotubularins to phosphoinositides, and the PTP domain contains catalytic activity, we further determined whether PtdIns(3)P is required for locating MTM-1 to the cell membrane by examining the MTM-1::GFP expression pattern in *vps-34(lf)* mutants in which the production of PtdIns(3)P is largely blocked [54]. We found that the plasma membrane localization of MTM-1 was not obviously affected in *vps-34(h797)* mutants (Figure S5C). Moreover, no significant difference in the localization of MTM-1::GFP was observed in either *piki-1(ok2346)* mutants or *vps-34(h797);piki-1(ok2346)* double mutants (Figure S5C). Therefore, neither PI3Ks nor substrates of MTM-1 seem to be required for its plasma membrane localization.

### Inactivation of MTM-1 increases the accumulation of PtdIns(3)P on intracellular vesicles

**Figure 8:**
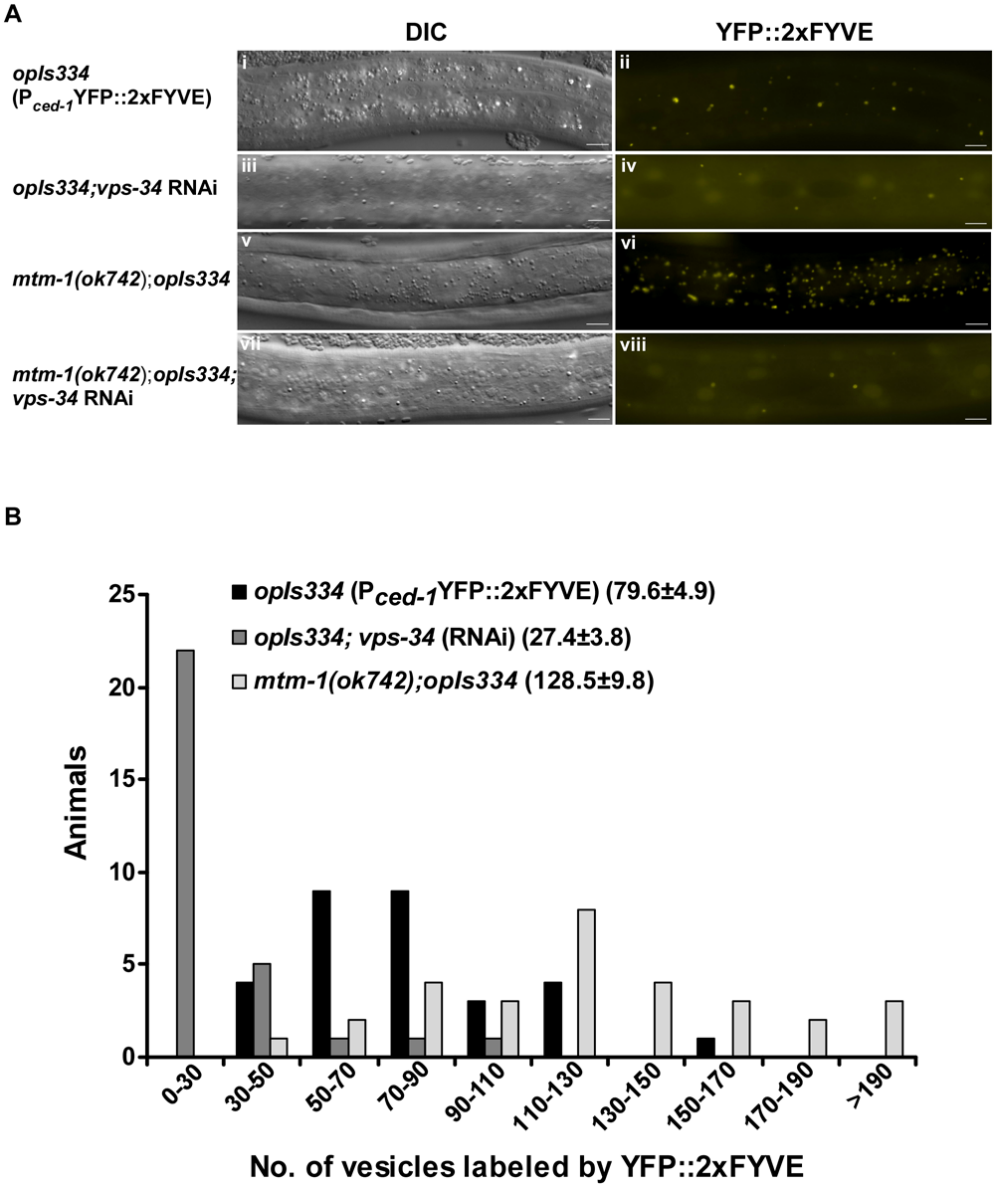
MTM-1 negatively regulates the level of PtdIns(3)P on intracellular vesicles. (A) The localization and accumulation of PtdIns(3)P were examined in hypodermal cells using a YFP::2xFYVE probe. *mtm-1(ok742)* larvae (v, vi) contained significantly higher numbers of YFP-positive vesicles than wild type (i, ii), whereas inactivation of PI3K VPS-34 by RNAi greatly reduced the PtdIns(3)P level in both wild type (iii, iv) and *mtm-1(ok742)* mutants (vii, viii). Images were captured using a fixed exposure time of 550 ms for wild type and *mtm-1(ok742)* mutants and 1500 ms for animals treated with *vps-34* RNAi. Bars: 5 µm (B) Quantification of data shown in (A). 30 L2 larvae from each strain were quantified as described in Materials and Methods. The *y*-axis shows the number of animals that contain YFP::2xFYVE-positive vesicles within a specific range as shown on the *x*-axis. The numbers in parenthesis indicate the average number of vesicles positive for YFP::2xFYVE (±s.e.m).

Because MTM-1 is a lipid phosphatase, its negative role in cell corpse engulfment might be achieved by regulating the level of PtdIns(3)P. To test this hypothesis and assess whether *mtm-1* can negatively regulate PtdIns(3)P levels in *C. elegans*, we monitored the localization and accumulation of PtdIns(3)P in both wild type and *mtm-1(ok742)* mutants using a YFP::2xFYVE probe which specifically binds PtdIns(3)P on both endosomes and phagosomes [44],[54]. Compared to wild-type animals, there were significantly more YFP::2xFYVE-positive vesicles in *mtm-1(ok742)* larvae, and the number of positive vesicles was greatly reduced when *vps-34* activity was inhibited (Figure 8A). This indicates that MTM-1 may modulate the level of PtdIns(3)P on intracellular vesicles. We then quantified YFP::2xFYVE labeling in hypodermal cells which highly express MTM-1 and can act as engulfing cells. In wild-type animals, we observed an average of 79 YFP::2xFYVE-positive vesicles, which was significantly reduced to 27 by treatment with *vps-34* RNAi (Figure 8B). This indicates that *vps-34* is responsible for generating PtdIns(3)P on these vesicles. In contrast, over 66% of *mtm-1(ok742)* worms contained more than 110 vesicles positive for YFP::2xFYVE. The average number of YFP-2xFYVE-positive vesicles in *mtm-1(ok742)* animals was 128, which is 62% more than in wild type, suggesting that MTM-1 negatively regulates the vesicular accumulation of PtdIns(3)P (Figure 8B).

## Discussion

### Why is a negative regulation of engulfment required?

The rapid and efficient phagocytosis of apoptotic cells is crucial for maintaining homeostasis as well as regulating the immune responses. Therefore, it seems to be counterintuitive to think that engulfment needs to be down-regulated. In fact, phagocytosis of apoptotic cell not only clears dead cells but promotes cell killing. For example, macrophages taking up apoptotic cells will release FasL and promote Fas-mediated apoptosis of monocytes and neutrophils for quick resolution of inflammation [52]. It has also been reported that macrophages are involved in tissue remodeling by promoting apoptosis in vertebrates [56]–[58]. Furthermore, genetic studies in *C. elegans* showed that blocking engulfment enhances the survival of cells triggered to initiate programmed cell death [59],[60]. Hence, it is conceivable that uncontrolled engulfment may lead to unexpected cell deaths under certain circumstances and an inhibitory mechanism may protect cells from inappropriate death. In addition, negative regulation of engulfment may also contribute to the correct targeting of apoptotic cells by phagocytes. In contrast to apoptotic cells that expose “eat me” flags on the surface, living cells exhibit “don't eat me” signals, such as CD47 and CD31, in order to be discriminated from dead cells [52],[61]. Although intracellular pathways transducing this type of signal have not been identified, it is possible that negative regulators are involved in mediating “don't eat me” signals within engulfing cells and in inhibiting the engulfment of normal healthy cells.

### MTM-1 negatively regulates cell corpse engulfment through CED-5/12/10

Although many genes are known be involved in apoptotic cell clearance, very few of them play a negative role. In mammalian macrophages, the small GTPase RhoA and its effector Rho-kinase were shown to negatively regulate the engulfment of apoptotic cells [62],[63]. Recently, *C. elegans* ABL-1 kinase was reported to inhibit cell corpse engulfment and DTC migration through its interacting protein ABI-1 [64]. However, the underlying mechanisms whereby these genes negatively regulate engulfment are not clear.

In order to gain more insight into the negative regulation of phagocytosis, we performed an RNAi screen to search for negative regulators of cell corpse engulfment and identified *C. elegans* myotubularin MTM-1, which was previously reported to play a role in endocytosis. Our genetic and cell biological analyses indicate that inactivation of MTM-1 promotes cell corpse engulfment, while overexpression of MTM-1 results in accumulation of cell corpses, suggesting that MTM-1 negatively regulates the engulfment of apoptotic cells. Given that inactivation of *mtm-1* reduced the number of cell corpses in strong loss-of-function mutants of *ced-1*, *ced-6*, *ced-7* and *ced-2* but not *ced-5*, *ced-12* or *ced-10*, *mtm-1* likely functions through the CED-5/CED-12/CED-10 complex. We still observed a significant reduction in cell corpses when *mtm-1* was inhibited in *ced-2(n1994);mig-2(mu28)* double mutants, which suggests that MIG-2 and CED-2 do not mediate all the inputs into the CED-10 pathway and an additional branch may exist in parallel to both of them. Since overexpression of MTM-1 failed to enhance the engulfment phenotype in *ced-2*, *ced-5*, *ced-12* or *ced-10* mutants, we prefer a model in which *mtm-1* functions downstream of *mig-2* and *ced-2* to negatively regulate the CED-5/CED-12/CED-10 complex rather than a model where *mtm-1* acts in a parallel pathway which requires CED-10 function for engulfment. Recently, ABL-1 kinase was reported to inhibit both cell corpse engulfment and DTC migration by acting in a parallel pathway to the two known engulfment pathways. Moreover, the functions of CED-5, CED-12 and CED-10 were required for its inhibition of engulfment but not DTC migration [64]. As both ABL-1 and MTM-1 negatively regulate cell corpse engulfment through CED-5, CED-12 and CED-10, it will be interesting to test the genetic interaction between them.

### MTMs play non-redundant roles in the engulfment of apoptotic cells

In *C. elegans*, 5 MTMs (MTM-1, 3, 6, 9 and 5) have been identified based on sequence homology. They belong to 5 different subgroups with *mtm-5* and *mtm*-*9* encoding catalytically inactive phosphatases and probably perform non-redundant functions like mammalian MTMs [36]. In agreement with this notion, we found that MTM-1, but not other MTMs, plays a negative role in cell corpse engulfment. Interestingly, *mtm-3(lf);mtm-6(lf)* double mutants contain significantly higher numbers of cell corpses than wild type and they also enhance the cell corpse phenotype of *ced-1(e1735)* mutants, suggesting that *mtm-3* and *mtm-6* may play a redundant role to either promote cell corpse clearance or affect cell death activation. Since *mtm-6* has only been implicated in regulating an ARF-6- and RME-1-dependent endocytic pathway together with *mtm-9*, whereas the functions of *mtm-3* are mostly unknown, it will be important to determine whether these MTMs are directly involved in cell death processes and if so, which specific steps they regulate and how their activities are coordinated.

### MTM-1 may down-regulate cell corpse engulfment by modulating plasma membrane PtdIns(3)P levels

Myotubularins are lipid phosphatases that specifically dephosphorylate PtdIns(3)P or its metabolite PtdIns(3,5)P_2_ [35]. Our findings that MTM-1 acts as a negative regulator of cell corpse engulfment dependent on its lipid phosphatase activity suggest that 3-phosphoinositides may act as signaling molecules during engulfment. Given that both the class III PI3K *vps-34* and the class II PI3K *piki-1* are required for reducing cell corpses by *mtm-1* RNAi and that loss of *mtm-1* function increases the vesicular accumulation of PtdIns(3)P in vivo, PtdIns(3)P likely serves as a substrate of MTM-1 during engulfment. Although PtdIns(3)P is enriched in endocytic compartments [54], we found that MTM-1 is mainly localized to plasma membrane. Although both the phosphoinositide-binding domain PH-GRAM and the catalytic domain PTP are required for membrane association of MTM-1, PtdIns(3)P seems to be dispensable for its membrane localization because MTM-1 still localizes to the plasma membrane in *vps-34(lf)*, *piki-1(lf)* or *vps-34(lf);piki-1(lf)* double mutants, in which PtdIns(3)P generation is probably blocked [54]. This observation also excludes the possibility that MTM-1 is recruited to plasma membranes through direct interaction with VPS-34 or PIKI-1. Therefore, MTM-1 may associate with plasma membranes through its interactions with other phosphinositides or protein partners such as VPS34 adaptor protein which binds hMTM1 on endosomes [65]. Consistent with our observations, several human MTMs were also reported to localize to plasma membranes although their substrates PtdIns(3)P and PtdIns(3,5)P_2_ are concentrated on early and late endocytic compartments, respectively, which leads to the hypothesis that myotubularins may act to prevent accumulation of PtdIns(3)P or PtdIns(3,5)P_2_ at inappropriate compartments [21],[35]. In the case of MTM-1, it is possible that MTM-1 coordinates with PI3Ks VPS-34 and PIKI-1 to maintain an appropriate level of PtdIns(3)P on the plasma membrane for the internalization of cell corpses. In agreement with this hypothesis, we found that MTM-1 transiently associates with extending pseduopods and nascent phagosomes at a similar stage to the phagocytic receptor CED-1 during engulfment.

Although PtdIns(3)P is a proven critical regulator of early endosomal traffic and phagosome maturation, its involvement in cell corpse engulfment has not been reported. We propose that PtdIns(3)P acts as a positive signal for engulfment based on our findings that (i) *mtm-1* antagonizes cell corpse internalization, which requires both its lipid phosphatase activity and the functions of PI3Ks VPS-34 and PIKI-1, (ii) MTM-1 localizes to the plasma membrane, functions in engulfing cells and clusters around cell corpses during engulfment, and (iii) MTM-1 negatively regulates the vesicular accumulation of PtdIns(3)P in vivo. Interestingly, DOCK180, the mammalian homolog of CED-5, binds directly to PtdIns(3,4,5)P_3_ through its DHR1 domain in vitro and translocates to the cell membrane in response to PtdIns(3,4,5)P_3_ production in NIH3T3 cells [66]. Since the Class I PI3K AGE-1 which generates PtdIns(3,4,5)P_3_ appears not to be required for cell corpse engulfment in *C. elegans*, it is possible that CED-5/CED-12 GEF is recruited to the plasma membrane by PtdIns(3)P to activate CED-10/Rac, and that MTM-1 acts antagonistically to terminate the signal and release the complex after engulfment. Alternatively, PtdIns(3)P may bind and facilitate the nucleotide exchange of CED-10/Rac during engulfment, similar to the way in which PtdIns(4,5)P_2_ promotes Rho and Rac activation under certain conditions [67].

### The function of MTM-1 in cell corpse engulfment may be conserved from *C. elegans* to mammals

Myotubularin family phosphatases are conserved amongst all eukaryotic organisms, but their cellular functions are not well understood. Our finding that overexpression of human MTM1 can efficiently rescue the cell corpse phenotype of *mtm-1(lf)* mutants suggests that the role of MTM-1 in cell corpse engulfment is likely conserved from worms to humans and that similar mechanisms may also be used in mammals to regulate the removal of apoptotic cells.

We found that MTM-1 acts as a lipid phosphatase to negatively regulate the engulfment of apoptotic cells through CED-10/Rac, which raises the interesting question of whether MTM-1 is similarly involved in other CED-10/Rac-mediated cellular processes by regulating PtdIns(3)P. Intriguingly, we found that *mtm-1* seems to play a positive instead of a negative role in the migration of DTCs, a process which also requires the activity of CED-5, CED-12 and CED-10. MTM-1 may therefore play distinct roles in different CED-10/Rac-mediated processes. However, we currently do not know whether the function of MTM-1 in DTC migration is also mediated by PtdIns(3)P and, if so, whether the opposite roles of MTM-1 in cell corpse engulfment and DTC migration are caused by distinct effects of PtdIns(3)P on CED-10/Rac in these two processes. On the other hand, loss of myotubularin has been found to cause muscle defects in human, mouse and zebrafish [17],[46],[68], but the underlying mechanism is not understood. Since we have identified a genetic link between MTM-1 and CED-10/Rac in cell corpse engulfment, it will be interesting to test whether misregulation of Rac GTPase might be relevant to the muscle defect caused by lack of myotubularin.

## Materials and Methods

### *C. elegans* strains

Strains of *C. elegans* were cultured at 20°C using standard procedures [69]. The N2 Bristol strain was used as the wild-type strain. Mutations used are described in *C. elegans* II [70] unless otherwise indicated. Linkage group I (LGI): *ced-1(e1735)*, *vps-34(h797)*, *dpy-5(e61)*, *unc-13(e450am)*, *mtm-1(ok742)* (this study), *ced-12(n3261)* [12], *hT2(bli-4(e937);let-?(q782)qIs48/sep-1(e2406)* (Wormbase:www.wormbase.org). LGII: *rrf-3(pk1426)* [71]. LGIII: *ced-6(n2095), ced-6(qx17)* (this study and see below), *ced-7(n2094)*, *mtm-6(ok330)* (this study, Wormbase: www.wormbase.org). LGIV: *ced-2(n1994), ced-5(n1812), ced-10(n3246), ced-10(n1993)*. LGX: *mig-2(mu28)* [72], *piki-1(ok2346)* and *mtm-5(ok469)* (this study, Wormbase: www.wormbase.org). *smIs34* (P*_ced-1_ced-1::gfp*) and *smIs95* (P*_hsp_annexin v::mrfp*) were provided by Dr. Ding Xue (University of Colorado, CO). *opIs334* (P*_ced-1_*YFP::2xFYVE) was a gift from Dr. K. S. Ravichandran (Univ. of Virginia, Charlottesville, VA) and Dr. M. O. Hengartner (Univ. of Zurich, Zurich, Switzerland) [44].

Other strains carrying integrated arrays used in this study are listed below:

*qxIs156* (P*_hsp_*MTM-1), *qxIs210* (P*_hsp_*MTM-1(C378S)), *qxIs197* (P*_mtm-1_*MTM-1::GFP), *qxIs66* (P*_ced-1_*GFP::RAB-7), *qxIs40* (P*_ced-1_*ACT-5::GFP), *qxIs60* (P*_ced-1_*LMP-1::GFP). The *vps-34*-deficient strain, *vps-34(h797)*, is maintained as *dpy-5(e61)vps-34(h797)*; *qxEx* [*vps-34*(+); P*_sur-5_sur-5::gfp*]. Non-green embryos (*dpy-5(e61)vps-34(h797))* were scored as *vps-34(lf)* and green embryos that carry the *vps-34(+)* transgene were scored as wild type for *vps-34*.

*qx17* is a recessive mutation isolated from a forward genetic screen for additional regulators of cell corpse engulfment and was mapped to linkage group III very close to *ced-6*. A complementation test between *qx17* and *ced-6(n2095)* was performed and a similar engulfment phenotype was observed in the double heterozygote, indicating that *qx17* is an allele of *ced-6*. We determined the sequence of *ced-6* in *qx17* mutants and identified a T to A transition that results in a premature stop codon after Glu 475 and generates a truncated CED-6 protein lacking the last 17 amino acids at the C-terminus.

### Quantification of cell corpses, cell death events, and cell corpse duration

Somatic cell corpses were directly visualized by differential interference contrast (DIC) microscopy as highly refractile button-like objects distinct from normal living cells [73],[74]. *C. elegans* embryos were mounted on agar pads in M9 and viewed using a 100× Plan-Neofluar DIC objective on an Axioimager M1 microscope (Carl Zeiss, Inc.). The number of cell corpses was quantified in the head region of living embryos either at the six different embryonic stages (bean/comma, 1.5-fold, 2-fold, 2.5-fold, 3-fold and 4-fold) for a time-course analysis or at the 4-fold embryonic stage as described before [75]. To measure the duration of cell corpses, four-dimensional microscopy (4D) analysis was performed at 20°C as described before with some modifications for *vps-34(h797);piki-1(ok2346)* mutants [9]. Since *vps-34(lf)* causes embryonic lethality and most *vps-34(h797)* mutant embryos die before the 4-fold embryonic stage, it is maintained as *dpy-5(e61)vps-34(h797)*; *qxEx* [*vps-34*(+); P*_sur-5_sur-5::gfp*]. To monitor the cell corpse duration in *vps-34(h797);piki-1(ok2346)* mutants, only non-green embryos which developed normally until the 2.5- or 3-fold stage were followed and quantified. To examine the internalization of cell corpses, apoptotic cells were first identified by their refractile disk-like morphology using Nomarski DIC microscopy and then different fluorescent markers that associate with cell corpses at different stages of engulfment were examined. To monitor the occurrence of embryonic cell death, embryos at the 2–4 cell stage were mounted on agar pads and images in a 30 µm z series (0.75 µm/section) were captured every 1.5 minutes for 8 h using a Zeiss Axioimager M1 microscope (Carl Zeiss, Inc.). Images were processed and viewed using Axiovision Rel 4.5 software (Carl Zeiss, Inc.).

### RNAi and genome-wide RNAi screen

The genome-wide RNAi screen was performed as described before with some modifications [76]. Briefly, adult hermaphrodites of *ced-1(e1735);rrf-3(pk1426)* were bleached on NGM plates without OP50. The hatched larvae were washed off with M9 and added to individual RNAi plates with 30 larvae per plate (*C. elegans* RNAi library, Geneservice, UK). Cell corpses were scored in the progeny at the 4-fold embryonic stage. For *mtm-1* RNAi by feeding, gravid adults of the indicated strains (P0) were picked and bleached on either control (pPD129.36-gfp or pPD129.36 for examining MTM-1::GFP expression) or *mtm-1* RNAi plates (I-6C09) and the number of embryonic cell corpses or the expression of MTM-1::GFP was scored in the F2 generation. To quantify the DTC migration defect, L4 larvae of the F2 generation were aged 24 h before examination. *mtm-1* RNAi caused 14% and 40% embryonic lethality in wild type and the RNAi hypersensitive mutant *rrf-3(pk1426)*, respectively, while 7% of wild-type and 29% of *rrf-3(pk1426)* embryos died when treated with *gfp* RNAi. For *mtm-1* RNAi by injection, a double-stranded RNA (dsRNA) of *mtm-1* (436–2489 bp of Y110A7A.5) was synthesized in vitro and injected into *ced-1(e1735);qxIs197* animals, which carry an integrated array of MTM-1::GFP controlled by the *mtm-1* promoter (P*_mtm-1_*MTM-1::GFP). Embryonic cell corpses and expression of MTM-1::GFP were both examined 24 h post injection. For *mtm-3* and *mtm-9* RNAi, double-stranded RNAs (dsRNA) were synthesized (*mtm-3*: 5812–6435 bp of T24A11.1a; *mtm-9*: 1583–2031 bp of Y39J10A.3a) and injected individually or in combination. Embryonic cell corpses were quantified 24 h post injection.

### Heat-shock experiments

Young adults were moved to fresh nematode growth medium (NGM) plates and cultured at 20°C for 12 h before they were incubated at 33°C for 1 h (+HS) or left at 20°C without heat-shock treatment (−HS), followed by recovery at 20°C for 1.5 h. Adult worms were removed and embryos were incubated at 20°C and scored for the number of cell corpses 5 to 10 h after treatment.

### Confocal microscopy

A Zeiss LSM 510 Pascal inverted confocal microscope with 488, 514, 633 lasers (Carl Zeiss Inc.) was used to capture fluorescent images which were processed and viewed using LSM Image Browser software. Time-lapse imaging of CED-1::GFP, GFP::MTM-1 and CED-1::mCHERRY was performed as described [45]. Briefly, *C. elegans* embryos at the pre-comma or comma stage were mounted on agar pads and images in a 20–25 z series (1.0 µm/section) were captured every 1 min, 1.5 min or 2 min for 120 min using a Zeiss LSM 510 Pascal inverted confocal microscope (Carl Zeiss, Inc.). Images were processed and viewed using LSM Image Browser software.

### Examination of PtdIns(3)P level by YFP::2xFYVE probe

To examine the level of PtdIns(3)P using the YFP::2xFYVE probe, L2 larvae of wild type, surviving *mtm-1(ok742)* mutants derived from *hT2/mtm-1(ok742)* worms, and *vps-34* RNAi-treated wild type or *mtm-1(ok742)* mutants were mounted on agar pads. Nomarski and fluorescent images of the midbody region of L2 larvae in a 20 z series (1.0 µm/section) were captured using a fixed exposure time with a Zeiss Axioimager A1 equipped with epifluorescence and an Axiocam monochrome digital camera. The exposure time was 550 ms for wild type and *mtm-1(ok742)* mutants, but 1500 ms for animals treated with *vps-34* RNAi because only very faint YFP::2xFYVE signal can be observed at 550 ms in these worms due to significant reduction of PtdIns(3)P accumulation caused by loss of *vps-34* function. Serial optical sections were analyzed and the numbers of YFP::2xFYVE-positive vesicles were quantified in hypodermal cells in a region of approximately 88 µm×18 µm. At least 30 animals were quantified in each strain.

### Plasmid construction

PCR primer sequences are shown in Table S4. To generate P*_mtm-1_mtm-1::gfp* , a 3.9 kb genomic fragment of *mtm-1* including a 0.8 kb promoter region was amplified by PCR from WRM0617dG02 using primers PQL121/120 and cloned into the pPD95.77 vector through its Bam HI and Sma I sites. A 3.1 kb fragment containing the full-length genomic sequence of the *mtm-1* gene was PCR-amplified from WRM0617dG02 using primers PWDL108/109 and cloned into both pPD49.78 and pPD49.83 through the Nhe I-Kpn I sites to generate P*_hsp_mtm-1*. The same *mtm-1* genomic fragment was also amplified by primers PWZ215/PWDL109 and cloned into P*_ced-1_gfp* vector through the Kpn I site to generate P*_ced-1_gfp::mtm-1*. The C378S mutation was introduced into pPD49.83-mtm-1 by site-directed mutagenesis using primers PQL148/149 (QuickChange; Stratagene, USA) and re-cloned into both pPD49.78 and pPD49.83 via their Nhe I and Kpn I sites. To construct P*_ced-1_mtm-1* and P*_egl-1_mtm-1*, the full-length cDNA of *mtm-1* was amplified from a *C. elegans* cDNA library (Invitrogen, USA) by PWZ215/PWDL109 and cloned into both P*_ced-1_* and P*_egl-1_* vector through the Kpn I site. To generate P*_ced-1_*GFP::MTM-1(ΔGRAM), P*_ced-1_*GFP::MTM-1(ΔPTP) and P*_ced-1_*GFP::MTM-1(Δcoiled-coil), a 2.1 kb genomic fragment of *mtm-1* (ΔGRAM: 975–3072 bp) or a 1.7 kb *mtm-1* genomic sequence (1–1739 bp) (ΔPTP) or a 3 kb *mtm-1* fragment (Δcoiled-coil: 1–3000 bp) were PCR-amplified from WRM0617dG02 using primers PWZ291/PWDL109, PWZ215/PWZ151 and PWZ215/PWZ322, respectively, and cloned into the P*_ced-1_gfp* vector via its Kpn I site. To construct P*_hsp_*hMTM1, the full-length cDNA of human *MTM1* was amplified from a human cDNA library (Clontech, USA ) with primers PWZ384/385 and cloned into both pPD48.78 and pPD49.83 through the Nhe I-Kpn I sites. To generate P*_hsp_myri::mcherry*, mcherry was amplified from pAA65 [77] using primers PWZ421 (which contains a myristoylation signal) and PWZ427 and cloned into both pPD49.78 and pPD49.83 through the Kpn I site.

## Supporting Information

Figure S1 *mtm-1* RNAi treatments specifically inhibit the expression of *mtm-1*. (A) *mtm-1* RNAi treatments (either feeding with bacteria expressing *mtm-1* dsRNA or injecting in vitro-synthesized *mtm-1* dsRNA) result in reduction of cell corpse numbers and inhibition of MTM-1::GFP expression. RNAi experiments were performed as described in Materials and Methods. Cell corpses were scored at the 4-fold embryonic stage and are shown as mean±s.e.m. At least 15 embryos were scored for cell corpses and 40 embryos at the 4-fold embryonic stage were examined for expression of MTM-1::GFP. Representative pictures of MTM-1::GFP expression before and after *mtm-1* RNAi treatment are also shown. The exposure time of both pictures was 2000 ms. (B,C) The gene structures of *mtm-1* and *piki-1* are shown, with filled boxes representing the exons and thin lines indicating the introns. The arrows show the direction of the transcript. The gray bars below the genes indicate the position and size of the deletions in the *ok742* and *ok2346* mutant.
(1.05 MB TIF)

Figure S2 *mtm-1* RNAi accelerates internalization of the apoptotic cell C3. (A) DIC and fluorescence images of a wild-type embryo co-expressing CED-1::GFP (P*_ced-1_ced-1::gfp*) and a secreted Annexin V::mRFP under the control of heat-shock promoters (P*_hsp_annexin v::mrfp*) are shown. The apoptotic cell C3 (white arrow) and a posterior apoptotic cell (blue arrow) were labeled by both CED-1::GFP and Annexin V::mRFP. The ventral hypodermal cell that engulfs C3 is indicated by the arrowhead. Bars: 5 µm. (B,C) The formation and duration of the CED-1::GFP ring around C3 (arrowed) were followed in *ced-6(qx17)* mutants treated with either control (i–iv in B and i–v in C) or *mtm-1* RNAi (v–viii in B and vi–x in C). To monitor the formation of CED-1 rings, the “0 min” time point was set immediately prior to the appearance of trace amounts of CED-1::GFP around C3. To monitoring the duration of CED-1 rings, the “0 min” time point was set when a full CED-1::GFP ring was just visible. 13 C3 corpses were monitored and quantified for either formation or duration of CED-1::GFP rings (ix in B and xi in C). The numbers in parenthesis indicate average formation or duration times of CED-1::GFP rings (±s.e.m). Bars: 5 µm.
(2.95 MB TIF)

Figure S3 Inactivation of MTM-1 promotes cell corpse internalization. Clustering of CED-1::GFP around cell corpses (A) or the phagosomal association of GFP::RAB-7 (B) and LMP-1::GFP (C) was quantified in 1.5-fold stage embryos in the indicated strains. At least 15 embryos were scored in each strain. Error bars indicate s.e.m. Unpaired *t* tests were performed to compare the data derived from *mtm-1* RNAi-treated embryos with that from control animals. **P<0.0001, *P<0.01; all other points had *P* value>0.01.
(0.13 MB TIF)

Figure S4 Myotubularin is conserved in yeast, worms and humans. Protein sequence alignments of *C. elegans* MTM-1 (c.eMTM-1), human myotubularin (hMTM1) and yeast myotubularin (Ymr1p) are shown. Identical residues are in black and similar ones are in gray. Conserved motifs are boxed. The signature CX5R active site motif for the protein tyrosine phosphatase super-family is circled in red. The critical cysteine residue, which is changed to serine in the *C. elegans* MTM-1(C378S) mutant, is marked by a red arrowhead.
(0.50 MB TIF)

Figure S5 Plasma membrane localization of MTM-1 does not require the activities of PI3-kinases. (A) MTM-1::GFP is expressed in many different cell types. DIC and fluorescence images of wild-type animals expressing P*_mtm-1_mtm-1::gfp* are shown. MTM-1::GFP was seen in distal tip cells (i, ii), coelomocytes (iii, iv) and vulva cells (v, vi). Bars: 10 µm. (B) Both full-length and truncated GFP::MTM-1 were stably expressed in *C. elegans*. Lysates were prepared from 200 adult transgenic worms carrying P*_ced-1_*GFP::MTM-1, P*_ced-1_*GFP::MTM-1(δGRAM), P*_ced-1_*GFP::MTM-1(δPTP) or P*_ced-1_*GFP::MTM-1(δCC) and western blot analysis was performed using an anti-GFP antibody. Full-length GFP::MTM-1 (93 Kd) and the three GFP::MTM-1 truncations (δCC: 91 Kd, δGRAM: 76 Kd, δPTP: 65 Kd) were all expressed at the expected size. (C) DIC and fluorescence images of MTM-1::GFP in wild-type (i, ii), *vps-34(h797)* (iii, iv), *piki-1(ok2346)* (v, vi) and *vps-34(h797);piki-1(ok2346)* (vii, viii) embryos are shown. The plasma membrane localization of MTM-1::GFP is not affected in the loss-of-function mutants of PI3-kinases. Bars: 5 µm.
(2.85 MB TIF)

Table S1 Inactivation of MTM-1 affects the migration of DTCs.
(0.04 MB DOC)

Table S2 The lipid phosphatase activity and conserved domains of MTM-1 are important for its function in cell corpse engulfment.
(0.04 MB DOC)

Table S3 Other MTMs do not play redundant roles with MTM-1 in cell corpse engulfment.
(0.04 MB DOC)

Table S4 Primers used for plasmid construction.
(0.04 MB DOC)

Video S1 The clustering of CED-1::GFP in a *ced-6(qx17)* embryo treated with *mtm-1* RNAi. The formation of a CED-1::GFP ring around a dying cell in a *mtm-1* RNAi-treated *ced-6(qx17)* embryo is shown. The cell corpse followed is indicated by an arrow. The frames were collected every 1 min and displayed every 1 sec. Selected images are shown in Figure 4A.
(0.35 MB AVI)

Video S2 The clustering of CED-1::GFP in a *ced-6(qx17)* embryo treated with control RNAi.The formation of a CED-1::GFP ring around a dying cell in a control RNAi-treated *ced-6(qx17)* embryo is shown. The cell corpse followed is indicated by an arrow. The frames were collected every 1.5 min and displayed every 1 sec. Selected images are shown in Figure 4A.
(0.33 MB AVI)

Video S3 The duration of CED-1::GFP around dying cell in a *ced-6(qx17)* embryo treated with control RNAi.The duration of the CED-1::GFP ring around a cell corpse in a control RNAi-treated *ced-6(qx17)* embryo is shown. The cell corpse followed is indicated by an arrow. The frames were collected every 1 min and displayed every 1 sec. Selected images are shown in Figure 4C.
(0.81 MB AVI)

Video S4 The duration of CED-1::GFP around dying cell in a *ced-6(qx17)* embryo treated with *mtm-1* RNAi.The duration of CED-1::GFP around a cell corpse in a *mtm-1* RNAi-treated *ced-6(qx17)* embryo is shown. The cell corpse followed is indicated by an arrow. The frames were collected every 1 min and displayed every 1 sec. Selected images are shown in Figure 4C.
(0.52 MB AVI)
