# Supplementary figures and images for: Caenorhabditis elegans Myotubularin MTM-1 Negatively Regulates the Engulfment of Apoptotic Cells
Source: PLoS Genet. 2009 Oct 9;5(10):e1000679. doi: 10.1371/journal.pgen.1000679 (PMC2751444; doi:10.1371/journal.pgen.1000679)

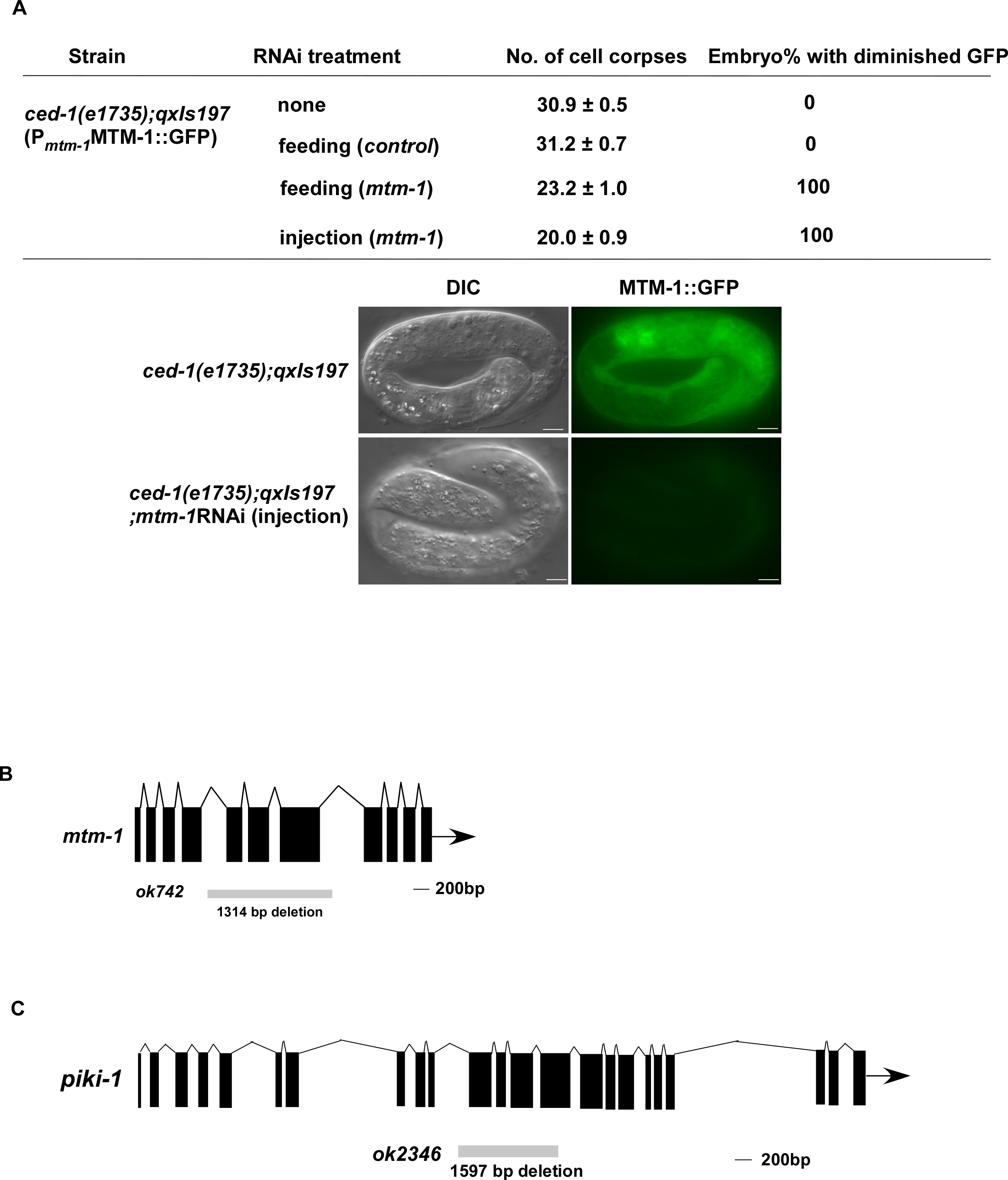

Supplement: Figure S1 — mtm-1 RNAi treatments specifically inhibit the expression of mtm-1. (A) mtm-1 RNAi treatments (either feeding with bacteria expressing mtm-1 dsRNA or injecting in vitro-synthesized mtm-1 dsRNA) result in reduction of cell corpse numbers and inhibition of MTM-1::GFP expression. RNAi experiments were performed as described in Materials and Methods. Cell corpses were scored at the 4-fold embryonic stage and are shown as mean±s.e.m. At least 15 embryos were scored for cell corpses and 40 embryos at the 4-fold embryonic stage were examined for expression of MTM-1::GFP. Representative pictures of MTM-1::GFP expression before and after mtm-1 RNAi treatment are also shown. The exposure time of both pictures was 2000 ms. (B,C) The gene structures of mtm-1 and piki-1 are shown, with filled boxes representing the exons and thin lines indicating the introns. The arrows show the direction of the transcript. The gray bars below the genes indicate the position and size of the deletions in the ok742 and ok2346 mutant. (1.05 MB TIF) [file pgen.1000679.s001.tif]

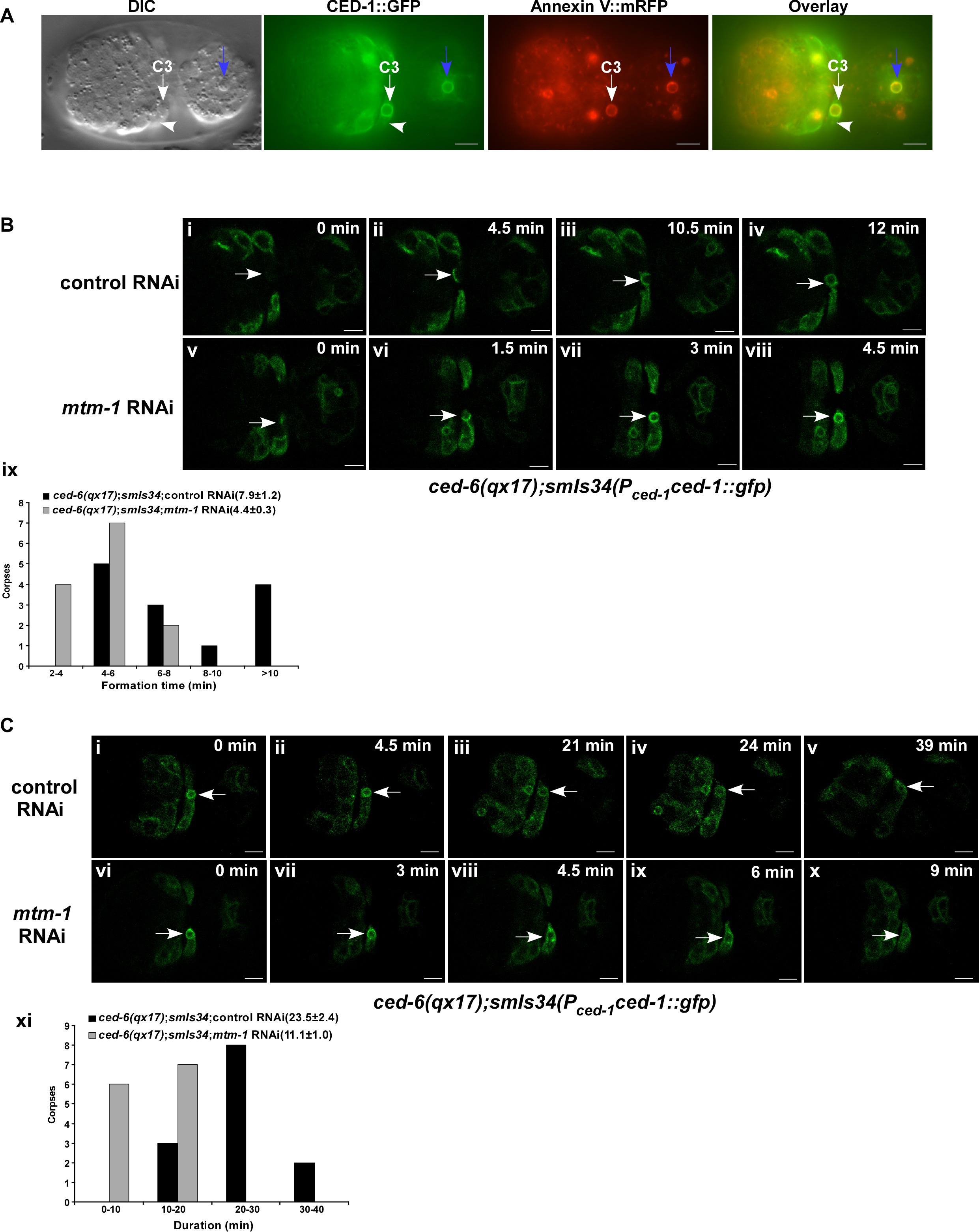

Supplement: Figure S2 — mtm-1 RNAi accelerates internalization of the apoptotic cell C3. (A) DIC and fluorescence images of a wild-type embryo co-expressing CED-1::GFP (Pced-1ced-1::gfp) and a secreted Annexin V::mRFP under the control of heat-shock promoters (Phspannexin v::mrfp) are shown. The apoptotic cell C3 (white arrow) and a posterior apoptotic cell (blue arrow) were labeled by both CED-1::GFP and Annexin V::mRFP. The ventral hypodermal cell that engulfs C3 is indicated by the arrowhead. Bars: 5 µm. (B,C) The formation and duration of the CED-1::GFP ring around C3 (arrowed) were followed in ced-6(qx17) mutants treated with either control (i–iv in B and i–v in C) or mtm-1 RNAi (v–viii in B and vi–x in C). To monitor the formation of CED-1 rings, the “0 min” time point was set immediately prior to the appearance of trace amounts of CED-1::GFP around C3. To monitoring the duration of CED-1 rings, the “0 min” time point was set when a full CED-1::GFP ring was just visible. 13 C3 corpses were monitored and quantified for either formation or duration of CED-1::GFP rings (ix in B and xi in C). The numbers in parenthesis indicate average formation or duration times of CED-1::GFP rings (±s.e.m). Bars: 5 µm. (2.95 MB TIF) [file pgen.1000679.s002.tif]

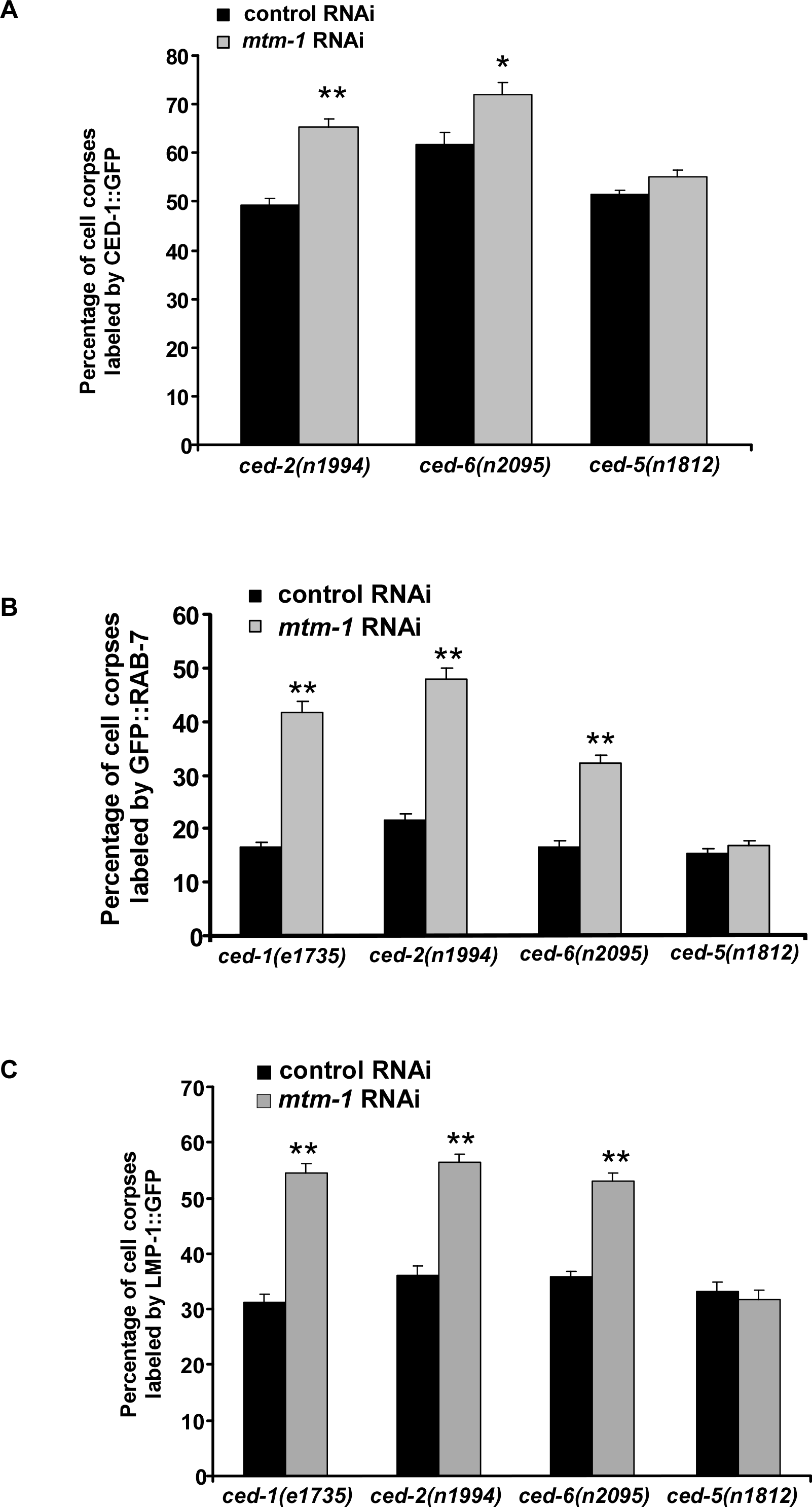

Supplement: Figure S3 — Inactivation of MTM-1 promotes cell corpse internalization. Clustering of CED-1::GFP around cell corpses (A) or the phagosomal association of GFP::RAB-7 (B) and LMP-1::GFP (C) was quantified in 1.5-fold stage embryos in the indicated strains. At least 15 embryos were scored in each strain. Error bars indicate s.e.m. Unpaired t tests were performed to compare the data derived from mtm-1 RNAi-treated embryos with that from control animals. **P<0.0001, *P<0.01; all other points had P value>0.01. (0.13 MB TIF) [file pgen.1000679.s003.tif]

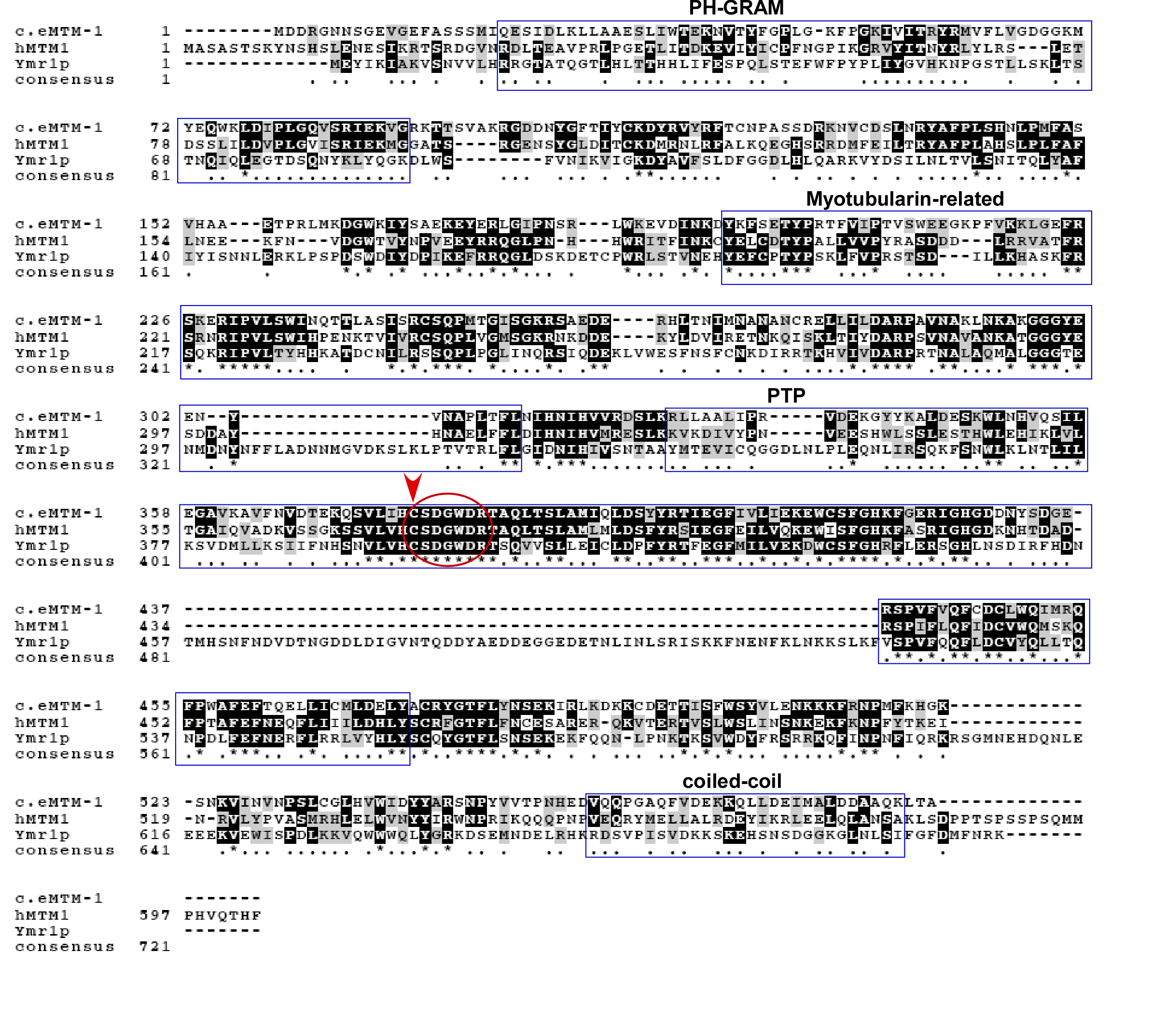

Supplement: Figure S4 — Myotubularin is conserved in yeast, worms and humans. Protein sequence alignments of C. elegans MTM-1 (c.eMTM-1), human myotubularin (hMTM1) and yeast myotubularin (Ymr1p) are shown. Identical residues are in black and similar ones are in gray. Conserved motifs are boxed. The signature CX5R active site motif for the protein tyrosine phosphatase super-family is circled in red. The critical cysteine residue, which is changed to serine in the C. elegans MTM-1(C378S) mutant, is marked by a red arrowhead. (0.50 MB TIF) [file pgen.1000679.s004.tif]

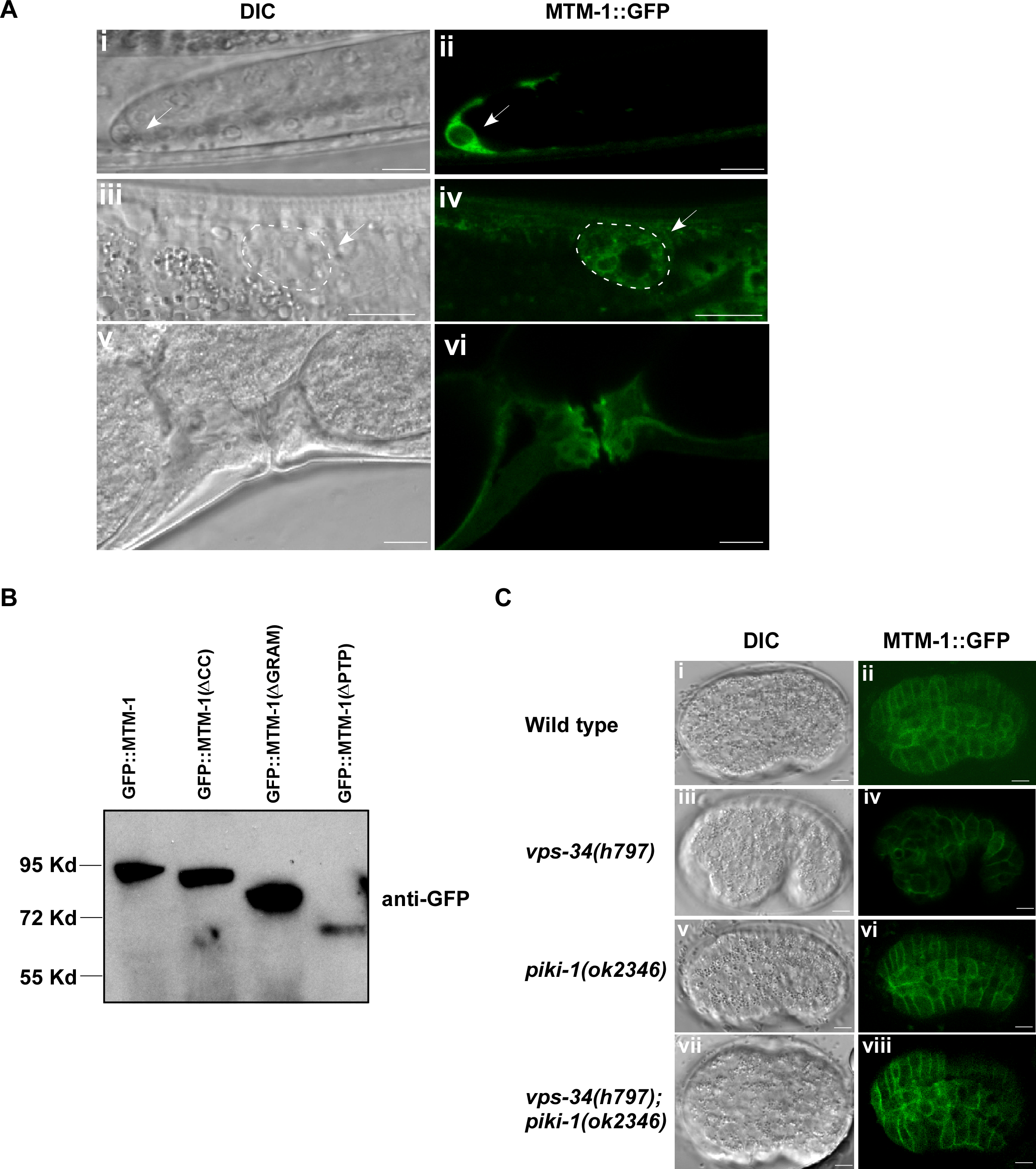

Supplement: Figure S5 — Plasma membrane localization of MTM-1 does not require the activities of PI3-kinases. (A) MTM-1::GFP is expressed in many different cell types. DIC and fluorescence images of wild-type animals expressing Pmtm-1mtm-1::gfp are shown. MTM-1::GFP was seen in distal tip cells (i, ii), coelomocytes (iii, iv) and vulva cells (v, vi). Bars: 10 µm. (B) Both full-length and truncated GFP::MTM-1 were stably expressed in C. elegans. Lysates were prepared from 200 adult transgenic worms carrying Pced-1GFP::MTM-1, Pced-1GFP::MTM-1(δGRAM), Pced-1GFP::MTM-1(δPTP) or Pced-1GFP::MTM-1(δCC) and western blot analysis was performed using an anti-GFP antibody. Full-length GFP::MTM-1 (93 Kd) and the three GFP::MTM-1 truncations (δCC: 91 Kd, δGRAM: 76 Kd, δPTP: 65 Kd) were all expressed at the expected size. (C) DIC and fluorescence images of MTM-1::GFP in wild-type (i, ii), vps-34(h797) (iii, iv), piki-1(ok2346) (v, vi) and vps-34(h797);piki-1(ok2346) (vii, viii) embryos are shown. The plasma membrane localization of MTM-1::GFP is not affected in the loss-of-function mutants of PI3-kinases. Bars: 5 µm. (2.85 MB TIF) [file pgen.1000679.s005.tif]
