# Supplementary material for: Caenorhabditis elegans Myotubularin MTM-1 Negatively Regulates the Engulfment of Apoptotic Cells
Source: PLoS Genet. 2009 Oct 9;5(10):e1000679. doi: 10.1371/journal.pgen.1000679 (PMC2751444; doi:10.1371/journal.pgen.1000679)
Supplement: Table S1 — Inactivation of MTM-1 affects the migration of DTCs. (0.04 MB DOC) [file pgen.1000679.s006.doc]

**Table S1. Inactivation of MTM-1 affects the migration of DTCs.**

| **Genotype** | **% abnormal**  **gonad arms^*^** | **No. of gonad arms**  **scored** |
| --- | --- | --- |
| **N2*;control RNAi*** | **2.8** | **70** |
| **N2*;mtm-1 RNAi*** | **14** | **70** |
| ***rrf-3(pk1426);control RNAi*** | **1.4** | **70** |
| ***rrf-3(pk1426);mtm-1 RNAi*** | **17** | **72** |
| ***ced-2(n1994);control RNAi*** | **53** | **30** |
| ***ced-2(n1994);mtm-1 RNAi*** | **53** | **30** |
| ***ced-5(n1812);control RNAi*** | **57** | **30** |
| ***ced-5(n1812);mtm-1 RNAi*** | **63** | **30** |
| ***ced-12(n3216);control RNAi*** | **54** | **72** |
| ***ced-12(n3216);mtm-1 RNAi*** | **50** | **72** |
| ***ced-10(n3246);control RNAi*** | **47** | **30** |
| ***ced-10(n3246);mtm-1 RNAi*** | **40** | **30** |
| ***mig-2(mu28);control RNAi*** | **23** | **60** |
| ***mig-2(mu28);mtm-1 RNAi*** | **25** | **60** |
| ***ced-2(n1994);mig-2(mu28);control RNAi*** | **43** | **60** |
| ***ced-2(n1994);mig-2(mu28);mtm-1 RNAi*** | **37** | **60** |
| ***ced-1(e1735);ced-2(n1994);control RNAi*** | **42** | **60** |
| ***ced-1(e1735);ced-2(n1994);mtm-1 RNAi*** | **45** | **60** |
| ***ced-10(n1993);control RNAi*** | **23** | **132** |
| ***ced-10(n1993);mtm-1 RNAi*** | **42** | **132** |

Feeding RNAi treatment was performed as described in Materials and Methods.

^*^Abnormal gonad arms were scored based on the shape of the gonad in young adults. The most frequently observed defects are inappropriate turns and twists of gonad arms.
