# Supplementary material for: Caenorhabditis elegans Myotubularin MTM-1 Negatively Regulates the Engulfment of Apoptotic Cells
Source: PLoS Genet. 2009 Oct 9;5(10):e1000679. doi: 10.1371/journal.pgen.1000679 (PMC2751444; doi:10.1371/journal.pgen.1000679)
Supplement: Table S2 — The lipid phosphatase activity and conserved domains of MTM-1 are important for its function in cell corpse engulfment. (0.04 MB DOC) [file pgen.1000679.s007.doc]

**Table S2. The lipid phosphatase activity and conserved domains of MTM-1 are important for its function in cell corpse engulfment.**

| **Strain** | **Transgene** | **No. of cell corpses^2^** | **Rescue^3^** |
| --- | --- | --- | --- |
| ***ced-2(n1994)*** | **none** | **28.3 ± 0.7** | **N.A** |
| ***mtm-1(ok742);ced-2(n1994)* ^1^** | **none** | **17.3 ± 0.7** | **N.A** |
|  | **P*_hsp_*MTM-1^4^** | **29.3 ± 0.8** | **+** |
|  | **P*_hsp_*MTM-1(C378S) ^4^** | **17.1 ± 0.9** | **-** |
|  | **P*_hsp_*hMTM1^5^** | **L1 31.0 ± 0.8**  **L2 30.2 ± 0.9**  **L3 29.2 ± 0.8** | **+**  **+**  **+** |
|  | **P*_mtm-1_*MTM-1::GFP^4^** | **30.2 ± 0.7** | **+** |
|  | **P*_ced-1_*MTM-1^5^** | **L1 30.6 ± 0.6**  **L2 28.2 ± 0.8** | **+**  **+** |
|  | **P*_egl-1_*MTM-1^5^** | **L1 17.2 ± 0.9**  **L2 17.5 ± 0.8** | **-**  **-** |
|  | **P*_ced-1_*GFP*::*MTM-1^5^** | **L1 30.3 ± 0.9**  **L2 29.7 ± 0.7** | **+**  **+** |
|  | **P*_ced-1_*GFP*::*MTM-1 (ΔGRAM) ^5^** | **L1 18.0 ± 0.7**  **L2 17.1 ± 1.1** | **-**  **-** |
|  | **P*_ced-1_*GFP*::*MTM-1 (ΔPTP)^5^** | **L1 16.5 ± 0.6**  **L2 19.1 ± 0.8** | **-**  **-** |
|  | **P*_ced-1_*GFP*::*MTM-1 (ΔCC)^5^** | **L1 26.5 ± 1.4**  **L2 30.8 ± 0.9** | **+**  **+** |

Heat-shock experiments were performed as described in Materials and Methods.

^1^*mtm-1(ok742)* mutants were maintained as *hT2/mtm-1(ok742)* and non-hT2 embryos were scored as homozygotes of *mtm-1(ok742)*.

^2^Cell corpses were scored in the head region of 4-fold stage embryos and are shown as mean±s.e.m. At least 15 embryos were scored for each rescue experiment.

^3^Strains that exhibited rescue activity contained similar numbers of cell corpses to *ced-2(n1994)* mutants and are indicated as positive (+) for rescue. Strains that failed to show rescue activity contained the same number of cell corpses as *mtm-1(ok742);ced-2(n1994)* embryos and are indicated as negative (-) for rescue.

^4^Strains carrying an integrated array of MTM-1

^5^Strains carrying extra chromosomal arrays of MTM-1. At least 2 independent transgenic lines were scored for rescuing activity.
