# Supplementary material for: Caenorhabditis elegans Myotubularin MTM-1 Negatively Regulates the Engulfment of Apoptotic Cells
Source: PLoS Genet. 2009 Oct 9;5(10):e1000679. doi: 10.1371/journal.pgen.1000679 (PMC2751444; doi:10.1371/journal.pgen.1000679)
Supplement: Table S3 — Other MTMs do not play redundant roles with MTM-1 in cell corpse engulfment. (0.04 MB DOC) [file pgen.1000679.s008.doc]

**Table S3. Other MTMs do not play redundant roles with MTM-1 in cell corpse engulfment.**

| **Genotype** | **No. of cell corpses^1^** | **Changes** | ***p-* value^2^** |
| --- | --- | --- | --- |
| ***ced-1(e1735)*** | **31.7 ± 0.6** |  | **-** |
| ***ced-1(e1735);mtm-5(ok469)*** | **31.2 ± 0.6** | **No** | **0.6** |
| ***ced-1(e1735);mtm-6(ok330)*** | **31.5 ± 0.7** | **No** | **0.9** |
| ***ced-1(e1735);mtm-6(ok330);mtm-5(ok469)*** | **32.0 ± 0.6** | **No** | **0.7** |
| ***ced-1(e1735);mtm-3( RNAi*)** | **34.0 ± 0.7** |  | **0.01** |
| ***ced-1(e1735);mtm-9( RNAi*)** | **31.0 ± 0.6** | **No** | **0.4** |
| ***ced-1(e1735);mtm-3 (RNAi)mtm-6(ok330)*** | **41.3 ± 1.0** |  | **<0.0001** |
| ***ced-1(e1735);mtm-3 (RNA);mtm-5(ok469)*** | **33.5 ± 1.0** | **No** | **0.1** |
| ***ced-1(e1735);mtm-3 (RNAi)mtm-6(ok330);mtm-5(ok469)*** | **39.2 ± 1.0** |  | **<0.0001** |
| ***ced-1(e1735);mtm-6(ok330);mtm-9(RNAi);mtm-5(ok469)*** | **31.2 ± 0.7** | **No** | **0.6** |
| ***ced-1(e1735);mtm-3(RNAi)mtm-6(ok330);mtm-9(RNAi);***  ***mtm-5(ok469)*** | **39.3 ± 1.2** |  | **<0.0001** |
| **Wild type *(N2)*** | **8.6 ± 0.4** |  | **-** |
| ***mtm-3* *(RNAi)*** | **12.4 ± 0.5** |  | **<0.0001** |
| ***mtm-5(ok469)*** | **8.5 ± 0.4** | **No** | **0.9** |
| ***mtm-6(ok330)*** | **9.1 ± 0.5** | **No** | **0.4** |
| ***mtm-3*(*RNAi*);*mtm-5(ok469)*** | **10.5 ± 0.6** |  | **0.006** |
| ***mtm-3*(*RNAi*);*mtm-6(ok330)*** | **22.9 ± 1.0** |  | **<0.0001** |

RNAi experiments were performed as described in Materials and Methods.

^1^Cell corpses were scored in 4-fold stage embryos in strains carrying the *ced-1(e1735)* mutation, whereas 1.5/2-fold stage embryos were used for quantification in wild type or various *mtm(lf)* mutants without the *ced-1* mutation. At least 15 embryos were scored for each strain. Cell corpses are shown as mean±s.e.m.

^2^Unpaired *t* tests were performed to compare the data from *ced-1(e1735)* mutants with *ced-1;mtm(lf)* mutants or wild type animals with *mtm(lf)* worms.
