# Supplementary material for: Caenorhabditis elegans Myotubularin MTM-1 Negatively Regulates the Engulfment of Apoptotic Cells
Source: PLoS Genet. 2009 Oct 9;5(10):e1000679. doi: 10.1371/journal.pgen.1000679 (PMC2751444; doi:10.1371/journal.pgen.1000679)
Supplement: Table S4 — Primers used for plasmid construction. (0.04 MB DOC) [file pgen.1000679.s009.doc]

**Table S4. Primers used for plasmid construction.**

| **Primer** | **Sequence*** |
| --- | --- |
| **PQL121** | **GCggatcc ctagccaattatctatgccaaatgtac** |
| **PQL120** | **CGCCCGGG ggcggtcaatttttgagcagcgtc** |
| **PQL148** | **caatcagtattgattcatagttctgatggttgggatcgaac** |
| **PQL149** | **gttcgatcccaaccatcagaactatgaatcaatactgattg** |
| **PWDL108** | **CGACTAGT atggatgacagagggaacaatagtg** |
| **PWDL109** | **CGGGTACC ctaggcggtcaatttttgagcag** |
| **PWZ215** | **CGGGTACC atggatgacagagggaacaatagtg** |
| **PWZ151** | **CGGGTACC ctagtatcccttctcgtcgacacgtgga** |
| **PWZ291** | **GCGGTACC atgacacctagacttatgaaagatg** |
| **PWZ322** | **CGGGTACC ctaaaactgtgcgcctggctgttg** |
| **PWZ384** | **CGGCTAGCatggcttctgcatcaacttc** |
| **PWZ385** | **GCGGTACCtcagaagtgagtttgcacatgg** |
| **PWZ421** | **GC GGTACC atgggttcctgtattggaaaa gtctcaaagggtgaagaag** |
| **PWZ427** | **GC GGTACC ttacttatacaattcatccatg** |

* The capitalized sequences are restriction sites with two protective nucleotides to the 5’ and the letters in lower cases are sequences from corresponding genes. The nucleotide changes for generating point mutations are underlined in primers PQL148 and 149 and the sequence of myristoylation signal is boxed in primer PWZ421.
